# Amphiregulin in Fibrotic Diseases and Cancer

**DOI:** 10.3390/ijms26146945

**Published:** 2025-07-19

**Authors:** Tae Rim Kim, Beomseok Son, Chun Geun Lee, Han-Oh Park

**Affiliations:** 1 siRNAgen Therapeutics, Daejeon 34302, Republic of Korea; trkim@sirnagen.com (T.R.K.); bson@sirnagen.com (B.S.); 2 Department of Molecular Microbiology and Immunology, Brown University, Providence, RI 02912, USA; chun_lee@brown.edu; 3 Bioneer Corporation, Daejeon 34013, Republic of Korea

**Keywords:** amphiregulin, fibrosis, cancer, EGFR signaling, TGF-β, integrins, myofibroblasts, SAMiRNA

## Abstract

Fibrotic disorders pose a significant global health burden due to limited treatment options, creating an urgent need for novel therapeutic strategies. Amphiregulin (AREG), a low-affinity ligand for the epidermal growth factor receptor (EGFR), has emerged as a key mediator of fibrogenesis through dual signaling pathways. Unlike high-affinity EGFR ligands, AREG induces sustained signaling that activates downstream effectors and promotes the integrin-mediated activation of transforming growth factor (TGF)-β. This enables both canonical and non-canonical EGFR signaling pathways that contribute to fibrosis. Elevated AREG expression correlates with disease severity across multiple organs, including the lungs, kidneys, liver, and heart. The therapeutic targeting of AREG has shown promising antifibrotic and anticancer effects, suggesting a dual-benefit strategy. The increasing recognition of the shared mechanisms between fibrosis and cancer further supports the development of unified treatment approaches. The inhibition of AREG has been shown to sensitize fibrotic tumor microenvironments to chemotherapy, enhancing combination therapy efficacy. Targeted therapies, such as Self-Assembled-Micelle inhibitory RNA (SAMiRNA)-AREG, have demonstrated enhanced specificity and favorable safety profiles in preclinical studies and early clinical trials. Personalized treatment based on AREG expression may improve clinical outcomes, establishing AREG as a promising precision medicine target for both fibrotic and malignant diseases. This review aims to provide a comprehensive understanding of AREG biology and evaluate its therapeutic potential in fibrosis and cancer.

## 1. Introduction

Fibrotic diseases represent a significant global health burden, affecting over 800 million people worldwide and contributing to 45% of deaths in industrialized countries [1]. They represent a significant global health burden, characterized by the excessive accumulation of extracellular matrix (ECM) proteins that leads to progressive tissue scarring and organ dysfunction across multiple organ systems including the lungs, liver, kidneys, heart, and skin [1,2,3,4]. The pathological hallmark of fibrosis involves the persistent activation and proliferation of fibroblasts, which differentiate into myofibroblasts that produce excessive collagen and other ECM components, ultimately resulting in tissue stiffening, architectural distortion, and impaired organ function [5,6,7,8]. Despite the high morbidity and mortality associated with fibrotic disorders, therapeutic options remain limited, highlighting the urgent need to elucidate the molecular mechanisms underlying fibrogenesis and identify novel therapeutic targets [9,10,11].

Recent investigations have revealed the critical involvement of various growth factors and cytokines in orchestrating the complex cellular and molecular interactions that drive fibrotic processes. Among these mediators, amphiregulin (AREG) has emerged as a key regulator bridging tissue injury, inflammation, and repair mechanisms that can contribute to both physiological wound healing and pathological fibrosis [12,13,14,15,16]. AREG was first discovered and purified from the conditioned medium of MCF-7 human breast cancer cells treated with phorbol 12-myristate 13-acetate, revealing a unique bifunctional growth-modulating glycoprotein that exhibited the distinctive ability to both stimulate the proliferation of normal fibroblasts and keratinocytes while simultaneously inhibiting the growth of certain aggressive cancer cell lines, thus earning its name from this amphipathic regulatory function [17].

AREG belongs to the epidermal growth factor (EGF) family and functions primarily through the epidermal growth factor receptor (EGFR/ErbB1), yet possesses distinct binding characteristics and signaling properties that differentiate it from other family members [13,18]. Unlike EGF which exhibits high-affinity EGFR binding, AREG is a low-affinity EGFR ligand that induces tonic signaling without triggering receptor internalization [12,18]. This unique property allows AREG to preferentially induce the phosphorylation of EGFR at Tyr992, leading to the selective activation of the phospholipase C-γ (PLCγ) pathway rather than the canonical mitogen-activated protein kinase (MAPK) cascade [15,19]. The sustained PLCγ signaling has been shown to activate integrin complexes, particularly αvβ integrins, which play a crucial role in locally converting latent transforming growth factor (TGF)-β to its bioactive form [15]. This AREG-mediated mechanism represents a critical link between EGFR signaling and TGF-β activation, positioning AREG as a key determinant of local TGF-β function in fibrotic tissues.

AREG is encoded by a gene spanning approximately 10 kb that contains six exons, producing a 1.4 kb mRNA transcript that is translated into a 252-amino acid transmembrane precursor protein (pro-AREG) that undergoes proteolytic cleavage by metalloproteinases, particularly a disintegrin and metalloprotease (ADAM) 17, to release the mature soluble form consisting of 78–98 amino acids depending on the cleavage site [13,20,21]. This dual existence as both membrane-anchored and soluble forms enables AREG to function through multiple signaling modalities including autocrine, paracrine, and juxtacrine mechanisms, with the membrane-bound form capable of direct cell-to-cell signaling while the soluble form can act on distant targets [13,18,22]. Thus, AREG expression refers to transcription and translation of its precursor form, while secretion involves post-transcriptional cleavage by ADAM17. Early investigations emphasized AREG’s fundamental role in wound healing and tissue repair processes, where it serves as a critical mediator linking immune responses to tissue regeneration by promoting epithelial cell proliferation, migration, and differentiation while simultaneously modulating inflammatory responses [12,23,24,25]. While AREG plays essential roles in normal physiological wound healing and tissue homeostasis, that could protect pathologic fibrosis such as in endometrial fibrosis [26], accumulating evidence indicates its pathological involvement in chronic fibrotic diseases across multiple organ systems. In fibrotic conditions, sustained AREG expression creates a pathological feedback loop where activated fibroblasts and inflammatory cells continuously produce AREG, which in turn promotes further fibroblast proliferation and ECM synthesis through both canonical pathways and non-canonical EGFR signaling pathways involving αvβ integrin-mediated TGF-β activation [11,15,16,27,28]. This dual mechanistic pathway may explain why AREG contributes to EGFR inhibitor resistance and enhanced fibrosis in various pathological contexts [29,30].

The therapeutic potential of targeting AREG extends beyond simple EGFR signal blockade to include the disruption of the αvβ integrin–TGF-β axis, thereby simultaneously modulating both canonical and non-canonical fibrotic EGFR signaling pathways. Experimental evidence has demonstrated that AREG inhibition through genetic deletion, neutralizing antibodies, or siRNA significantly attenuates fibrosis development and progression across multiple organ systems [30,31,32,33]. Importantly, strategies targeting AREG have shown not only antifibrotic effects but also anti-tumor potential, presenting a promising therapeutic approach that could simultaneously target fibrosis and cancer progression.

The relationship between fibrosis and cancer is increasingly recognized as bidirectional, with shared mechanistic features that enable common therapeutic approaches. AREG inhibition has been shown to sensitize the fibrotic tumor microenvironment (TME) to chemotherapy, creating synergistic effects when combined with conventional anticancer drugs [13,18,29,34,35]. These findings highlight AREG’s involvement in drug resistance, demonstrating its potential as a therapeutic target for fibrotic disorders and fibrosis-associated cancers, as well as a biomarker for disease progression.

We reviewed the current understanding of AREG’s structural features, molecular mechanisms, physiological functions, and pathological roles in fibrotic diseases. We focus on AREG’s unique structural characteristics and signaling properties that distinguish it from other EGFR ligands, with particular emphasis on its dual capability to activate both canonical and non-canonical EGFR signaling pathways involving αvβ integrin–TGF-β interactions. We discuss AREG’s roles in the fibrosis of various organs, including pulmonary, hepatic, renal, cardiac, and cutaneous fibrosis, highlighting common pathological mechanisms as well as tissue-specific functions. Furthermore, we analyze the interplay between fibrosis and cancer, examining how AREG contributes to cancer progression within fibrotic microenvironments and its potential role in chemoresistance. Finally, we assess emerging therapeutic strategies targeting AREG and its signaling pathways, such as monoclonal antibodies, small-molecule inhibitors, and siRNA. We discuss their potential benefits, limitations, and ongoing clinical developments.

## 2. Mechanism of Action of AREG

AREG is a low-affinity ligand for the EGFR, distinguishing itself from high-affinity ligands such as EGF through its unique signaling pattern and downstream effects. Unlike EGF, which induces transient EGFR activation, AREG promotes sustained EGFR signaling with the preferential phosphorylation of the Tyr992 residue, a critical feature that contributes to EGFR tyrosine kinase inhibitor (TKI) resistance [12,15,19,36,37]. Resistance to EGFR-TKIs is multifactorial, involving both primary and acquired mutations in the EGFR gene, the activation of bypass signaling pathways, and limited drug uptake into target cells or tissues [12,38,39,40]. AREG contributes to EGFR-TKI resistance through bypass signaling via integrin αvβ-mediated TGF-β activation and the evasion of apoptosis through Ku70 acetylation and BAX inhibition [41,42]. Additionally, AREG’s tonic signaling does not induce receptor internalization, prolonging downstream pathway activation such as PLCγ and extracellular signal-regulated kinase (ERK)/MAPK cascades [37]. These findings align with the reported EGFR-TKI resistance mechanisms involving bypass pathways, impaired drug uptake, and tumor cell plasticity. This sustained signaling leads to the prolonged activation of downstream pathways, particularly ERK/MAPK cascades that drive persistent cellular activation and enhanced proliferation [43,44].

In fibrotic diseases, this prolonged EGFR activation results in the continuous stimulation of fibroblasts and epithelial cells, promoting excessive ECM production and tissue remodeling [16,45,46]. Moreover, AREG functions as a key amplifier of EGFR signaling, capable of integrating signals from other low-affinity EGFR ligands at the receptor level, thereby orchestrating a complex signaling network that drives pathological processes such as tissue fibrosis and tumorigenesis [34,47,48].

Beyond canonical EGFR signaling, AREG also exerts its profibrotic effects through non-canonical EGFR signaling pathways, notably via interaction with αvβ integrins. This interaction activates latent TGF-β, a master regulator of fibrosis, creating a critical crosslink between EGFR and TGF-β pathways [15,16,49]. Through this αvβ integrin-mediated TGF-β activation, AREG promotes epithelial–mesenchymal transition (EMT), a fundamental process in fibrogenesis and cancer metastasis [28,50]. It is notable that, unlike other low-affinity EGFR ligands, AREG uses both canonical and non-canonical EGFR signaling pathways, positioning AREG as a central mediator of both intrinsic and acquired resistance mechanisms in EGFR-driven diseases [29,51,52,53].

AREG expression exhibits distinct cellular patterns across fibrotic and tumor tissues. While AREG is produced by various immune cells, including macrophages, T cells, and type 2 innate lymphoid cells (ILC2s), it is particularly overexpressed in aberrant basaloid cells that contribute to disease pathogenesis [12,54,55]. In idiopathic pulmonary fibrosis (IPF), based on data mining through the IPF Cell Atlas portal (www.ipfcellatlas.com, accessed on 18 March 2025) and related studies, these aberrant basaloid cells represent a transitional epithelial phenotype derived from alveolar type 2 epithelial cell transdifferentiation, serving as key drivers of progressive fibrosis through sustained AREG production [30,56,57,58]. Similarly, cancer-associated fibroblasts and certain tumor epithelial cells exhibit elevated AREG expression, establishing a profibrotic and pro-tumorigenic microenvironment [29,59,60]. Additionally, bone marrow-derived CD11c^+^ cells have been identified as significant contributors of AREG in pulmonary fibrosis models [61]. This cell-specific expression pattern creates a complex intercellular communication network, where AREG-producing cells instruct neighboring cells to enhance ECM production and tissue remodeling [28,62,63].

Targeting AREG represents a promising therapeutic strategy for fibrotic diseases and cancer, particularly in contexts where EGFR-TKIs show limited efficacy. As a key driver of sustained EGFR activation and an integrator of signals from other low-affinity EGFR ligands, AREG serves as a central node in pathological signaling networks [12,16,34,48,64]. Novel approaches such as Self-Assembled-Micelle inhibitory RNA (SAMiRNA)-based therapies designed to silence AREG expression have shown efficacy in experimental models of renal and pulmonary fibrosis [31,32,33]. Furthermore, antibody-based therapies targeting AREG or targeting AREG shedding by ADAM17 antibody have demonstrated anti-tumor efficacy in preclinical models [65,66,67,68]. The therapeutic potential of AREG inhibition extends beyond direct effects on EGFR signaling, as it may also disrupt the αvβ integrin–TGF-β axis, addressing both canonical and non-canonical profibrotic EGFR signaling pathways [15,16,69,70]. We conducted a thorough review of the published studies on fibrosis occurring in various organs.

## 3. AREG and Fibrotic Diseases

### 3.1. Lung Fibrosis

IPF is a chronic, progressive, and ultimately fatal interstitial lung disease characterized by aberrant wound healing, excessive ECM deposition, and the irreversible scarring of the lung parenchyma. The disease typically presents progressive dyspnea, dry cough, and declining lung function, with a median survival of 3–5 years from diagnosis in the absence of treatment [71,72]. While the etiology remains incompletely understood, IPF is believed to result from complex interactions between genetic susceptibility, environmental exposures, and aging-related cellular dysfunction [73,74]. Currently approved antifibrotic therapies, including nintedanib and pirfenidone, have demonstrated modest efficacy in slowing disease progression but do not reverse established fibrosis or significantly improve survival rates [71,75,76]. These limitations, coupled with considerable side effects and variable patient responses, underscore the urgent need for more effective therapeutic approaches [77,78,79].

Emerging evidence indicates that AREG plays a crucial role in the pathogenesis of lung fibrosis and may serve as a prognostic biomarker. Multiple studies have demonstrated that elevated AREG levels correlate with disease severity and poor clinical outcomes in IPF patients. AREG levels are significantly elevated in various biological samples from patients with IPF and other pulmonary diseases compared to healthy controls, including circulation, bronchoalveolar lavage fluid, and sputum. These increased AREG levels correlate with lung function decline and mortality risk [30,80,81]. Moreover, single-cell RNA sequencing studies have revealed persistently high AREG expression in intermediate alveolar stem cells, suggesting that AREG may serve as a marker for transitional cell states that contribute to aberrant repair processes [30,82].

The profibrotic effects of AREG in lung fibrosis are mediated through multiple cellular sources and signaling pathways. Key populations include intermediate alveolar type 2 cells, which exhibit sustained AREG expression and contribute to failed regeneration [30,58,83]; bone marrow-derived CD11c^+^ cells, including dendritic cells and macrophages that accumulate in fibrotic lungs and secrete high levels of AREG [61,84]; ILC2s that produce AREG and regulate alveolar epithelial cell transdifferentiation [85]; and AREG-producing pathogenic memory T helper (Th) 2 cells that instruct eosinophils to secrete osteopontin, contributing to the airway fibrotic response and highlighting the complex immunopathology of AREG-mediated fibrosis [55,86]. AREG activates fibroblasts via EGFR, triggering downstream pathways such as phosphoinositide 3-kinase (PI3K)/Akt, MAPK/ERK, and Smad, which promote fibronectin, collagen, and ECM deposition [27,28]. AREG directly stimulates fibroblast proliferation, migration, and differentiation into myofibroblasts, further exacerbating tissue remodeling and fibrosis [12,16,27,69,87]. TGF-β, a key upstream regulator, induces AREG expression in epithelial cells, creating a feedforward loop that amplifies fibrosis. This persistent AREG signaling transforms physiological repair into pathological fibrosis. Additionally, AREG sustains a feedforward loop involving integrin-αv and TGF-β signaling, perpetuating fibroblast activation and ECM remodeling [15,16,88,89].

The identification of AREG as a key mediator in pulmonary fibrosis has prompted the development of targeted therapeutic strategies aimed at selectively inhibiting AREG signaling while avoiding the broad toxicities associated with general EGFR or TGF-β pathway inhibition. Several preclinical studies have demonstrated the efficacy of AREG-specific approaches in attenuating lung fibrosis. We demonstrated that SAMiRNA-targeting AREG (SAMiRNA-AREG) effectively reduced fibrotic markers and improved lung function in bleomycin-induced fibrosis models [33]. Similarly, AREG-targeting siRNA significantly reduced pulmonary fibrosis in TGF-β transgenic mice, with decreased collagen deposition and preserved lung architecture [87]. In vitro studies also demonstrated that AREG silencing effectively inhibited TGF-β-induced fibrotic responses in lung epithelial cells [28]. Anti-AREG monoclonal antibodies have shown reduced fibroblast activation and ECM production in vitro [30]. Areg-KO mice exhibit diminished fibrotic responses, validating its central role [30,61,86]. Unlike the broad effects of EGFR and TGF-β inhibitors, AREG-specific therapies minimize systemic toxicity while disrupting pathogenic signaling, offering a precision medicine strategy [15,30,90,91,92,93]. Notably, AREG gene expression varies with genetic background and immune system status, indicating that screening for AREG overexpression in patients before treatment could enhance the success rate of AREG inhibitors in IPF therapy [45]. AREG is a critical component in fibrotic cascades, where its persistent expression drives inappropriate repair processes. This makes it a promising target for stopping disease progression. AREG-targeting therapeutics may be a disease-modifying drug for IPF. Further clinical investigation is anticipated.

### 3.2. Kidney Fibrosis

Kidney fibrosis represents a common pathological outcome in chronic kidney disease (CKD), acute kidney injury (AKI), and diabetic kidney disease (DKD), characterized by persistent inflammation and progressive scarring that leads to significant renal dysfunction, increased morbidity, and mortality. These conditions share hallmark features including ECM accumulation, fibroblast activation, and tubular atrophy, ultimately resulting in irreversible tissue damage [21,94,95,96]. Despite advances in understanding the pathophysiology of renal fibrosis, current treatment options remain limited, with no definitive antifibrotic therapy available [97,98]. This significant unmet clinical need has driven research into novel therapeutic targets, among which AREG has emerged as a promising candidate due to its central role in mediating inflammatory and fibrotic responses in kidney disease [21,48,99,100].

Recent clinical studies have established AREG as a potential biomarker for kidney disease progression, with elevated levels detected in kidney biopsy tissues, serum, and urine samples from patients with CKD, AKI, and DKD [21,101,102]. These AREG levels correlate significantly with the degree of renal function decline, supporting its utility as both a diagnostic and prognostic indicator [21,31]. Notably, Osakabe and colleagues demonstrated that AREG concentrations in patients with CKD showed a strong association with disease severity [101], while the Chronic Renal Insufficiency Cohort study have demonstrated that urinary AREG, unlike serum levels, may provide more direct insights into kidney-specific processes, suggesting its potential as a superior biomarker for kidney health and disease progression [102]. The emerging diagnostic potential of AREG extends to early-stage fibrosis detection and therapeutic response monitoring, offering a novel approach to assess disease progression before irreversible damage occurs.

Mechanistically, AREG plays a complex and tissue-specific role in kidney fibrosis. It is produced primarily by resident renal cells including proximal and distal tubule cells and podocytes, and AREG functions as a crucial mediator in recruiting macrophages and monocytes to injured kidney tissue, promoting pro-inflammatory cytokine release and exacerbating tissue damage [31,48,99,103,104,105,106]. During acute kidney inflammation, AREG exhibits the most robust upregulation among EGFR ligands at the mRNA level [21,48,100]. The signaling cascade involves upstream regulators such as ADAM17 and TGF-β1, which activate the EGFR-yes-associated protein 1–AREG axis, leading to increased α-smooth muscle actin (α-SMA) expression, collagen deposition, ECM remodeling, and EMT processes [21,46,107,108,109,110,111]. Importantly, AREG demonstrates a dual nature depending on its cellular source: leukocyte-derived AREG mediates protective effects and promotes tissue repair, while tissue-derived AREG from resident kidney cells contributes to inflammation and fibrosis [12,31,48,99,112]. This dichotomy highlights the complexity of AREG signaling in renal pathophysiology and underscores the importance of targeted therapeutic approaches.

Emerging therapeutic strategies specifically targeting AREG offer promising alternatives to broad EGFR inhibition, potentially mitigating kidney fibrosis while minimizing adverse effects [31,48,99,113,114,115]. Preclinical studies employing various AREG-targeted approaches, including SAMiRNA-AREG, Areg-KO mouse models, and anti-AREG antibodies, have demonstrated significant reductions in fibrosis markers and improvements in renal outcomes [31,48,99,116]. For instance, Son et al. showed that SAMiRNA-AREG effectively ameliorated renal fibrosis through selective EGFR signal inhibition [31]. The natural product quercetin inhibits AREG/EGFR signaling-mediated renal tubular EMT and fibrosis in obstructive nephropathy [116]. Areg-specific therapies provide a more selective inhibition of the AREG-EGFR axis compared to conventional EGFR inhibitors and natural products, potentially reducing off-target effects while maintaining therapeutic efficacy [46,114]. These preclinical studies strongly support AREG-focused intervention as a novel and precise antifibrotic strategy, offering hope for more effective treatments for patients with CKD, AKI, diabetic nephropathy, and other fibrotic kidney disorders.

### 3.3. Liver Fibrosis

Fibrosis progression in the liver is caused by chronic injuries such as alcohol- or metabolic-associated fatty liver disease and hepatitis virus infection [117]. Liver fibrosis scores were correlated with the liver disease mortality, with 5-year all-cause mortality of advanced fibrosis being 14.9% [118,119]. Activated hepatic stellate cells (HSCs) in response to injury are the predominant source of myofibroblasts and fibrous scar in the liver. Liver fibrosis can be regressed by inactivating HSCs, inflammatory cells including macrophages, and fibrosis-driving signaling pathways [117]. Recent data have demonstrated that AREG is important for fibrogenesis in the liver by inducing HSCs to activated phenotype. In metabolic dysfunction-associated steatohepatitis (MASH) mouse models using fructose, palmitate, and cholesterol-rich (FPC) diet, AREG, produced by regulatory T cells, enhances the profibrotic transcription factors in HSCs via EGFR signaling [120]. This leads to HSC activation and liver fibrosis. Moreover, interleukin (IL)-6 produced by activated HSCs induced hepatic glucose intolerance, which eventually promoted gluconeogenesis by hepatocytes and elevated serum glucose levels [120]. These results suggest that inhibiting AREG in the liver with chronic liver injury might be critical for inhibiting liver fibrosis as well as regulating glucose levels. Other studies revealed that AREG increased proliferation of HSCs through mitogenic signaling pathways such as PI3K and p38 [121,122], whereas a protective role of AREG by inducing signal transducer and activator of transcription 1-dependent apoptosis of HSCs was also reported [123].

In March 2024, Resmetriom was approved by the U.S. Food and Drug Administration for treating noncirrhotic MASH with moderate-to-advanced liver fibrosis [124]. It is a thyroid hormone receptor-β agonist that enhances lipid metabolism and reduces liver fat. The approval of Resmetriom suggests that regulating an upstream signal relevant to generating or reversing liver fibrosis might be sufficient to improve disease condition. AREG is involved in signaling pathways that induce liver inflammation and fibrosis. An in vitro study with HepG2 cells showed that exogenous AREG treatment increased pro-inflammatory cytokine levels and activated nuclear factor-κB and MAPK signaling [125]. In addition, AREG induced cyclooxygenase-2 and inducible nitric oxide synthase, which are frequently overexpressed during inflammation [126,127]. Furthermore, the inhibition of AREG by reversion-inducing cysteine-rich protein with kazal motifs or quercetin (one of the plant flavonoids) ameliorated EGFR activation, contributing to reduced hepatic fibrosis progression [128,129]. Our group recently demonstrated that the inhibition of AREG using the SAMiRNA platform reduced hepatic lipid accumulation and attenuated fibrosis in a murine steatohepatitis model (data under review). These findings support the role of AREG in liver fibrogenesis and metabolic dysfunction. Taken together, these results show that targeting AREG is a feasible approach for liver fibrosis treatment.

At an initial phase of identifying the role of AREG in the liver, researchers found that AREG participated in liver regeneration [24,130,131] and fibrogenesis in the carbon tetrachloride (CCl_4_)-administered animals [132,133,134]. Additional studies helped to find out how increased AREG could lead to excessive liver fibrosis, as mentioned above. However, it should be considered that different animal models could give conflicting results on the role of AREG in the liver fibrosis. In the mice fed with the FPC diet, AREG produced by regulatory T cells promoted liver fibrosis [120], but regulatory T cell-derived AREG was not pivotal for inducing pathological states in the CCl_4_ liver injury [135].

Cholestatic liver diseases are characterized by the impairment of bile acid metabolism or bile flow. The hepatic accumulation of bile acids accounts for liver fibrosis and cirrhosis [136]. In the murine cholestatic liver injury models, AREG induced protective responses and reduced the apoptosis of liver cells by bile acids [137]. On the contrary, AREG derived from hepatic mucosal-associated invariant T (MAIT) cells promoted ductular reaction, which is associated with bile duct hyperplasia, abnormal wound healing response, and liver fibrosis [138]. As liver MAIT cell levels are correlated with the survival of biliary atresia patients after surgery and histological fibrosis, targeting AREG in biliary atresia would be a potential therapeutic strategy. It was found that hepatic AREG expression was elevated in cirrhotic patients resulting from MASH, primary sclerosing cholangitis, alcohol, autoimmune hepatitis, and hepatitis B and C viruses [139]. Further clinical investigation of AREG-targeting drug will prove the efficacy on liver fibrotic disease.

### 3.4. Cardiac Fibrosis

Cardiac fibrosis is a heterogeneous process influenced by diverse pathological triggers. In myocardial infarction, replacement fibrosis replaces necrotic cardiomyocytes with scar tissue to preserve structural integrity. In hypertension and pressure overload conditions, reactive interstitial fibrosis emerges due to chronic mechanical stress, characterized by excessive ECM deposition [140,141]. Regardless of its underlying mechanism, the pathological deposition of ECM proteins in the cardiac interstitium is a central feature of cardiac fibrosis. A large number of studies suggested that the extent of cardiac fibrosis was associated with poor prognoses, but it may indicate the activation of normal wound healing [140]. No drugs have been approved for treating cardiac fibrosis yet. It causes cardiac dysfunction in various diseases, including myocardial infarction, hypertrophic cardiomyopathy, and arrhythmogenesis [142,143,144,145,146]. As reviewed elsewhere, cardiac fibrosis is a consequence of wound healing response in the injured heart, but it can lead to lethal cardiac arrhythmias [146]. Accumulating evidence suggests that AREG is an important regulator of heart remodeling after injuries, as well as adverse effects such as the excessive proliferation of myofibroblasts. In murine cardiac pressure overload models, AREG produced by macrophages was necessary for maintaining homeostasis via hypertrophic and fibrotic response in cardiomyocytes and connexin 43 gap junction stabilization to regulate cardiac impulse conduction [147,148]. Hypoxia-inducible factor 2-α-dependent AREG expression alleviated myocardial ischemia–reperfusion injury [149]. In contrast, AKT/mammalian target of rapamycin signaling induced by AREG contributed to the development of myocardial hypertrophy and fibrosis, and heart failure after cardiac pressure overload [150]. The persistent and abnormally dysregulated expression and activation (ADAM17-mediated cleavage) of AREG-dependent EGFR signaling can promote myofibroblast proliferation and excessive cardiac fibrosis [151]. Humeres et al. revealed that Smad7 acted as a critical regulator of moderate remodeling in myofibroblasts of infarcted heart by inhibiting various TGF-β1 downstream signaling pathways [152]. In cardiac myofibroblasts, antifibrotic effects including decrease in ECM gene expressions by Smad7 and EGFR interaction was evident only in AREG-stimulated conditions [152]. This suggests that, in pathological conditions, Smad7 is defective, and AREG overexpression might result in unrestrained fibrosis generation through continuous EGFR activation. The capacity of AREG to induce other heart diseases requires further investigation, as AREG upregulation is related with the incidence of cirrhotic cardiomyopathy [153], hypertrophic cardiomyopathy [154], chronic rejection after heart transplantation [155], atrial fibrillation [156], and hypothermic cardiac deaths [157].

### 3.5. Intestinal Fibrosis

AREG helps to remodel intestine in inflammatory states such as colitis; however, this may lead to inflammatory bowel disease (IBD) or intestinal fibrosis if not appropriately regulated. AREG-expressing ILC2s in the gut exacerbated inflammation and disease severity in the dextran sodium sulfate mouse model of intestinal damage [158,159]. Cyclic adenosine monophosphate/protein kinase A, G-protein-coupled receptor 43, and B lymphocyte induced maturation protein 1 acted as upstream activators of AREG and EGFR signaling to induce tissue repair [160,161,162]. Contrary to the protective roles, AREG was highly expressed in intestinal biopsies of pediatric patients with IBD and severe graft-versus-host disease, which suggests that AREG staining could be used as a marker for severe inflammation [163]. Intestinal fibrosis is a frequent consequence of chronic inflammation in IBD, particularly Crohn’s disease [164,165]. Fibrotic complications could worsen the disease states by causing stricture formation, but antifibrotic therapy for treating intestinal fibrosis as the complication of Crohn’s disease has not been developed yet [166,167]. The mechanism of intestinal fibrosis development is an area of ongoing research, with an article by Yang et al. revealing that Th17 cell-derived AREG takes a central role [168]. Areg-KO mice showed harsher colitis than wild-type mice, but less severe intestinal fibrosis as evidenced by collagen and α-SMA gene expressions and collagen thickness in the colon. Moreover, the proliferation and migration of intestinal myofibroblasts isolated from Crohn’s disease patients was promoted by recombinant AREG treatment [168]. Remarkably, AREG expression in the intestinal biopsies of Crohn’s disease patients with fibrosis was elevated compared to tissues from patients without fibrosis. Collectively, these data suggest that AREG is involved in diverse functions in the intestines from regenerating damaged tissue to inducing chronic inflammation and fibrosis in a long-term fashion.

### 3.6. Radiation-Induced Fibrosis

Fifty-percent of all cancer patients including those with solid tumors receive radiation therapy [169]. Tissue fibrosis related to radiation might occur as an adverse effect of radiotherapy. Radiation injury elicits DNA damage and reactive oxygen species, which induces acute inflammation (minutes~days) and fibrosis (weeks~months) [170]. In our previous work, we demonstrated that total-body irradiation (TBI) to mice induced fibrosis in the kidneys [32]. AREG expression in the kidneys consistently increased 4 to 24 weeks after 6 Gy TBI. Fibrotic markers such as α-SMA and collagen type Iα1 were overexpressed at a later time point, namely, 24 weeks after TBI. AREG and fibrotic marker expressions were not significantly altered in other organs analyzed including the lungs, liver, intestines, and spleen. The knockdown of AREG using SAMiRNA-AREG reduced α-SMA expression in the proximal and distal tubules of the kidneys. Moreover, SAMiRNA-AREG diminished TBI-induced collagen accumulation in the cortex and medulla of the kidneys. These results suggest that inhibition of AREG might be a useful strategy to alleviate radiation-induced renal fibrosis [32]. Besides this study, the role of AREG in the fibrosis development upon irradiation has not been widely identified. Two individual studies showed protective effects of AREG at early response to irradiation using Areg-KO mice [160,171]. Intestinal regeneration in the 12 Gy TBI-exposed mice was not promoted without AREG at one week after TBI [160]. Radiation-induced AREG was primarily located in intestinal subepithelial myofibroblasts [160]. The other study adopted low-energy radiation such as ultraviolet B (UVB). Meulenbroeks et al. revealed that AREG derived from basophils was essential for the immune-suppressing function of UVB [171]. The immune response was induced by applying dinitrofluorobenzene to the mouse skin and left for 10 days. Therefore, these results suggest that AREG can take beneficial roles (i.e., remodeling and recovery) in the short term after irradiation, but the persistent activation of AREG may lead to chronic diseases including fibrosis.

### 3.7. Other Types of Fibrosis

Systemic sclerosis (SSc) is an autoimmune rheumatic disease that manifests uncontrolled fibrosis in the skin and internal organs [172]. In the bleomycin-induced skin fibrosis mouse models, AREG expression was consistently upregulated during fibrosis development [173]. Fibrosis and elevated cell proliferation in the dermis by bleomycin were not observed in the Areg-KO mice. Single-cell RNA sequencing results from lung explants of SSc-associated interstitial lung disease (SSc-ILD) indicated that the AREG expression of SSc-ILD in the natural killer (NK) cells was significantly higher than healthy controls [174]. Authors speculated about an interaction of AREG produced by the NK cells with SSc-ILD basal cells in which EGFR was upregulated. However, in the serum of SSc patients, AREG levels were not elevated compared to healthy controls [80]. The serum levels of AREG were higher in patients with idiopathic inflammatory myopathy (IIM), which is an autoimmune muscle disease [80]. IIM primarily features muscle weakness, but increasing research indicates that the development of interstitial lung diseases and pulmonary fibrosis are associated with IIM [175,176,177]. Therefore, antifibrotic drugs inhibiting AREG expression may alleviate lung diseases related to IIM [178].

Recent studies have revealed important roles for AREG in eosinophilic esophagitis (EoE) and esophageal fibrosis [179,180]. In the intranasal IL-33-induced EoE murine models, AREG derived by type 2 ILC2s and its activation of EGFR promoted esophageal epidermal thickening, abnormal proliferation, and fibrosis [179]. Notably, sole recombinant AREG administration to mice without IL-33 was sufficient to induce EoE phenotypes. AREG overexpression was also confirmed in the biopsies of human EoE patients [179]. In mouse EoE and esophageal fibrosis model that was exposed to long-term intranasal house dust mite antigen, Areg-KO reduced fibrotic responses [180]. Moreover, AREG-producing T cells colocalize with esophageal fibroblasts and AREG induces their proliferation. By performing single-cell RNA sequencing of esophagus from EoE patients, authors demonstrated that AREG-expressing Th2 cell infiltration was higher in the inflammatory and fibrotic lesions than control, and increased Th2 cell infiltration correlated with active state of esophageal fibrosis [180]. Taken together, these studies have revealed that AREG from rapid (ILC2s) and sustaining (Th2 cells) type 2 immunity was involved in the development of esophageal fibrosis, and AREG would be a promising target to alleviate the disease.

## 4. AREG and Cancer

AREG, a ligand of the EGFR, plays a crucial role in promoting cancer cell proliferation, survival, angiogenesis, invasion, metastasis, and the development of drug resistance [34]. It has been reported that AREG can induce the expression of programmed death-ligand 1 (PD-L1), contributing to immune evasion in the TME [35,181]. Elevated levels of circulating AREG have been identified in multiple cancer types, suggesting its potential utility as a serum biomarker for certain malignancies [182,183]. Importantly, AREG overexpression has been identified as a key mechanism underlying resistance to EGFR inhibitors such as gefitinib in non-small-cell lung cancer and other cancer types [42,182,184]. AREG also promotes oncogenic signaling in breast cancer, particularly in phosphatase and tensin homolog-null contexts, and can be secreted via exosomes to enhance cancer cell invasion capabilities [185,186].

Targeting AREG has emerged as a promising strategy to overcome EGFR inhibitor resistance and suppress tumor progression across various cancer types. RNA interference-mediated silencing of AREG, including the use of SAMiRNA-AREG, has demonstrated significant antifibrotic activity in preclinical models and is expected to yield anti-tumor effects, suggesting a promising therapeutic approach that potentially targets both fibrosis and cancer progression simultaneously [31,33]. Furthermore, AREG-neutralizing antibodies have been shown to inhibit tumor growth and metastasis in ovarian cancer models [187]. These therapeutic approaches suggest that blocking AREG activity can restore sensitivity to EGFR-targeted therapies in multiple cancer types including colorectal cancer [188,189], lung cancer [42], and pancreatic cancer [190]. The development of siRNA-based nucleic acid drugs targeting AREG represents a promising frontier in cancer therapeutics [41,191].

AREG, characterized as a low-affinity EGFR ligand, has distinct biological roles compared to high-affinity ligands such as heparin-binding EGF-like growth factor (HB-EGF) [12,37,40,43,44]. Notably, AREG is capable of activating TGF-β, a key regulator of fibrosis [15,16]. The inhibition of EGFR signaling disrupts HB-EGF-mediated homeostasis, which may contribute to fibrotic progression in patients receiving EGFR-targeted treatments [16]. The relationship between fibrosis and cancer is increasingly recognized as bidirectional, with shared mechanistic features suggesting common therapeutic approaches [60,192,193,194]. Therefore, suppressing AREG activity may be critical for mitigating fibrosis in EGFR inhibitor-treated cancer patients, as indicated by studies in multiple tissue contexts [16,45,195,196].

The inhibition of AREG has been shown to sensitize fibrotic TME to chemotherapy, suggesting a synergistic effect when combined with conventional anticancer drugs [34]. Combinational therapeutic strategies that include AREG-targeted inhibitors can reprogram the TME, enhancing anti-tumor efficacy [34,35,194,197]. Moreover, AREG produced by tumor-associated macrophages [60,198], cancer-associated fibroblasts [29,59,60,199], regulatory T cells [200,201], or dendritic cells [202], along with TGF-β, promotes collagen deposition and fibrosis in the tumor stroma [203]. These components establish a positive feedback loop that accelerates tumor progression and reinforces oncogenic signaling through EGFR-dependent pathways [28,204]. Importantly, the fibrotic TME creates a physical barrier that impedes drug delivery and contributes to drug resistance, further highlighting the importance of targeting AREG in cancer treatment [34,35,205,206].

Considering the pivotal role of TGF-β signaling in both fibrosis and tumor development, this pathway represents an attractive therapeutic target [207,208]. However, the pleiotropic and homeostatic functions of TGF-β limit its direct inhibition in clinical settings [70,209,210]. As such, targeting AREG presents a compelling alternative strategy to indirectly modulate TGF-β activity in the cancer microenvironment [15,16,70,211,212]. The development of combinational therapies incorporating AREG inhibitors holds great promise for remodeling the TME and enhancing therapeutic outcomes in various cancer types, including colorectal cancer [213,214], pancreatic cancer [190,215], head and neck cancer [29,216], ovarian cancer [181,187], and lung cancer [18,187,201,202,217,218]. By disrupting the AREG-mediated crosstalk between cancer cells and stromal components, these approaches may overcome the limitations of conventional cancer therapies and provide new avenues for clinical intervention.

A recently published paper highlighted that radiotherapy increased AREG expression in tumor tissues, which induced distant metastasis growth by reprogramming EGFR^+^ mononuclear phagocytes (MNPs) into an immunosuppressive phenotype [219]. AREG was one of the highly expressed genes induced by stereotactic body radiotherapy (SBRT) in 22 matched pre- and post-SBRT biopsies from irradiated metastases, and its expression correlated with the progression of unirradiated distant metastases in those patients. By utilizing spontaneous metastasis mouse models, authors revealed that local radiotherapy reduced the number of metastases but increased their size. Importantly, significantly smaller and fewer metastases following radiotherapy were observed in mice injected with Areg-KO cancer cells, compared to the corresponding wild-type cells. Radiotherapy-induced AREG targeted EGFR^+^ MNPs in the metastatic microenvironment, induced anti-inflammatory transcriptomic state in MNPs, and promoted phagocytosis resistance of tumor cells by increasing ‘don’t-eat-me’ CD47 signal. As reduced serum AREG levels correlated with longer progression-free survival of non-small-cell lung cancer patients treated with SBRT and immune checkpoint blockade, the potential of systemic AREG inhibition to delay tumor progression was evaluated [219]. Notably, AREG blockade by intravenous anti-AREG antibody injection enhanced the efficacy of anti-CD47 immunotherapy along with radiotherapy, effectively inhibiting metastatic proliferation. The antibody-mediated targeting of AREG has also shown promising results in decreasing both PD-L1-mediated immunosuppression and chemoresistance in preclinical tumor models [35]. These findings indicate that regulating AREG with anti-AREG antibodies or SAMiRNA-AREG enhances the effectiveness of radiotherapy alongside standard treatments.

## 5. Therapeutic Targeting of AREG: Preclinical and Clinical Trials for Human Application

Preclinical studies have shown that SAMiRNA-AREG mitigates fibrosis in the lungs, kidneys, and liver while demonstrating a favorable safety profile in both rodent and primate models. Its targeted mechanism and minimal immunogenicity make this approach a promising precision therapeutic for fibrotic and AREG-driven malignancies. The physiological expression profile of AREG provides strong evidence supporting the tolerability of AREG inhibition. According to the Human Protein Atlas [220], AREG expression is nearly undetectable in healthy human tissues except the placenta. This suggests that targeting AREG is unlikely to disrupt essential physiological functions. Moreover, studies using Areg-KO mice have shown normal reproductive capacity and life expectancy, reinforcing the safety of AREG suppression [221]. Additional studies with Areg-null mice demonstrated that AREG is not essential for bone anabolic action of parathyroid hormone [222] and, while AREG plays a role in mammary gland development [223] and liver regeneration [24], its absence does not cause significant physiological impairment. These findings provide strong preclinical justification for the safety of targeting AREG [30].

The therapeutic targeting of AREG aims to disrupt its pathological signaling involved in fibrosis and cancer. AREG can be inhibited at multiple levels: (1) by neutralizing its ligand activity using monoclonal antibodies, (2) by blocking AREG shedding through inhibition of ADAM17 protease, (3) by performing CRISPR-mediated gene editing, or (4) by downregulating its gene expression using RNA interference (RNAi).

Monoclonal antibodies targeting AREG directly neutralize its activity, blocking EGFR signaling and inhibiting tumor growth, as demonstrated by the AR37 antibody, which prolongs survival in preclinical cancer models and shows potential in treating fibrotic diseases due to its ability to reduce fibroblast activation [187]. However, these antibodies face challenges including high cost, complex manufacturing, limited tissue penetration in solid tumors, potential immune reactions, the risk of resistance development, and side effects such as hypertension and impaired wound healing; most anti-AREG and anti-ADAM17 antibodies remain in preclinical or early clinical development stages.

CRISPR-mediated gene editing offers the most durable suppression by permanently disrupting AREG or ADAM17 genes, leading to the sustained loss of function and robust anti-tumor effects in preclinical models with emerging epigenetic editing approaches enabling long-term silencing without permanent DNA alteration [224], potentially reducing off-target risks and improving safety for clinical translation. Yet, CRISPR approaches are limited by risks of off-target effects, delivery challenges, potential toxicity, low editing efficiency in some tissues, and ethical/regulatory hurdles; while gene editing is advancing rapidly with approved therapies for blood disorders, its application in solid tumors or organs is still in early clinical trials.

Among RNAi-based approaches, SAMiRNA represents a novel delivery platform that enhances the stability, bioavailability, and safety of siRNA therapeutics [225]. SAMiRNA-AREG specifically silences AREG mRNA, effectively reducing both EGFR-dependent and TGF-β signaling in fibrotic and tumor tissues.

The toxicological evaluation of SAMiRNA-AREG platform has been thoroughly conducted through four preclinical studies in both rodent and non-human primate models, revealing no significant adverse effects at various dose levels and durations. Kim et al. conducted the safety pharmacology studies of SAMiRNA-AREG and reported no abnormal findings in cardiovascular, respiratory, or central nervous system functions [225]. In a four-week repeated intravenous dose toxicity study in mice, Kim et al. demonstrated no significant toxicity at doses up to 300 mg/kg [226]. The genotoxicity evaluation by Kim et al. confirmed that SAMiRNA-AREG was non-mutagenic and non-clastogenic [227]. Most notably, a systemic toxicity and toxicokinetics study in cynomolgus monkeys following an intravenous injection revealed no adverse effects with repeated dosing [228]. Comprehensive toxicological assessments across these studies, including histopathology, hematology, clinical chemistry, and immunogenicity, showed no abnormal findings. The absence of organ toxicity or immune activation highlights the favorable safety profile of SAMiRNA-AREG in preclinical settings. Recent Phase 1a clinical trial of SAMiRNA-AREG (SRN001) in healthy human volunteers has yielded promising results (data under review). In a randomized, double-blind, placebo-controlled, single-ascending-dose Phase 1 clinical trial conducted in healthy participants, SRN001 demonstrated an acceptable safety profile across a dose range of 15 mg to 210 mg. No infusion-related reactions and no anti-drug antibodies were detected, indicating a low risk of immunogenicity and confirmed acceptable tolerability in humans. The favorable safety data for SAMiRNA-AREG provide a compelling rationale for its continued development as a promising therapeutic strategy for fibrotic diseases and cancer.

SAMiRNA-AREG represents a significant advancement in siRNA-based therapeutics, addressing many common challenges associated with siRNA drugs. siRNA drugs provide high target specificity and can effectively silence virtually any gene in the long term [191,229]. They are easier to design than small molecules, allow flexible routes of administration, have low dosing frequency, and can provide sustained effects with manageable safety profiles. However, conventional siRNAs are often unstable in vivo and susceptible to nuclease degradation. They are primarily delivered to the liver and may trigger innate immune responses [191,230,231,232,233]. Off-target effects and toxicity remain major barriers [191,234,235,236]. SAMiRNA-AREG overcomes these limitations through novel supramolecular structure [33]. The self-assembled micelle structure protects the siRNA from degradation while facilitating cellular uptake [31,237]. Notably, off-target analysis confirmed that SAMiRNA-AREG does not induce off-target gene expression and innate immune stimulation in preclinical and clinical studies. By selectively inhibiting AREG signaling, SAMiRNA-AREG provides a targeted method for treating pulmonary and other organ fibroses, while minimizing the side effects linked to broad EGFR and TGF-β inhibition [31,33]. The unique properties of the SAMiRNA platform, including enhanced stability, reduced immunogenicity, and efficient delivery to fibrotic and cancer tissues, overcome many of the traditional limitations of siRNA therapeutics [238,239]. These insights support SAMiRNA-AREG as a targeted and innovative approach for fibrosis and cancer therapy, with the potential to address a significant unmet medical need in patients with various fibrotic conditions.

## 6. Conclusions and Future Perspectives

**Figure 1:**
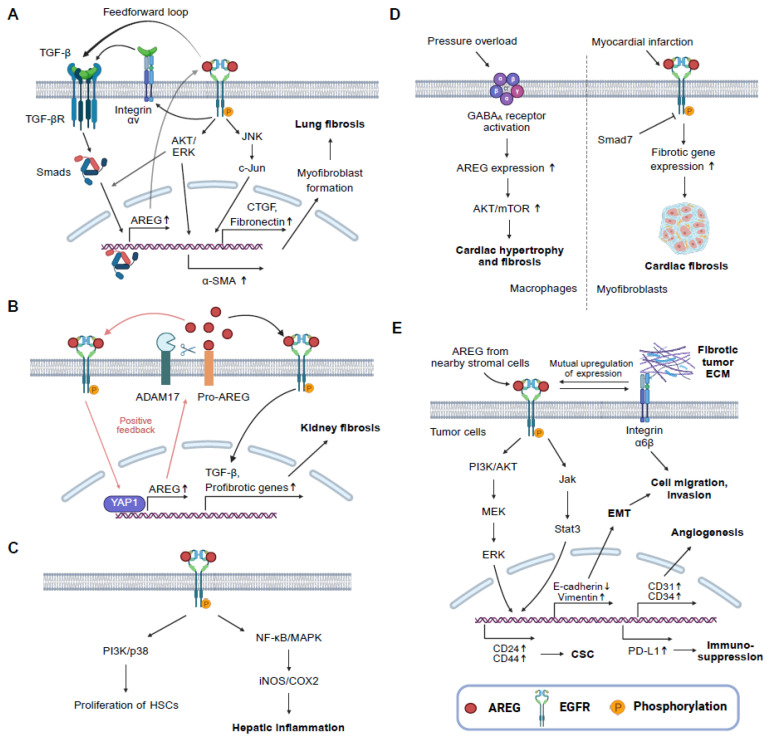
Signal transduction pathways of AREG inducing fibrosis and cancer. (**A**) AREG-mediated EGFR signaling stimulates AKT/ERK, JNK, and c-Jun to promote profibrotic protein expression such as CTGF, fibronectin, and α-SMA. AREG can be activated by TGF-β and can also activate TGF-β via integrin αv, which in turn induces AREG expression. The upward arrows (↑) next to gene or protein names indicate increased expression. (**B**) Kidney injury elevates ADAM17 and cleavage of pro-AREG in proximal tubules. It causes EGFR to be consistently activated, and YAP1 increases AREG transcription. Thus, AREG signaling can be amplified via a positive feedback loop. EGFR activation by AREG leads to profibrotic gene expressions. The upward arrows (↑) next to gene or protein names indicate increased expression. (**C**) AREG induced HSC proliferation and hepatic inflammation through mitogenic signaling such as PI3K, NF-κB, and MAPK. (**D**) In a cardiac pressure overload model, GABA_A_ receptor activity in macrophages is upregulated. Consequent AREG-induced AKT/mTOR signaling activation plays an important role in generating myocardial hypertrophy and fibrosis. Upon myocardial infarction, myofibroblast activation needs TGF-β-, ErbB2-, and AREG-EGFR-mediated signaling. Smad7 inhibits excessive remodeling in the infarcted heart. The upward arrows (↑) next to gene or protein names indicate increased expression or signal activation. (**E**) AREG that can be produced by DNA damage to nearby stromal cells activates EGFR and its downstream pathways in cancer cells. Moreover, AREG alters the transcriptomics of cancer cells—such as by promoting the expression of PD-L1, which facilitates immune evasion in the tumor microenvironment—thereby driving them to have immunosuppressive and aggressive phenotypes. AREG and integrin α6β are able to enhance expression mutually [240,241,242]. Activated integrins promote ECM deposition, contributing to form a fibrotic tumor. The upward (↑) or downward (↓) arrows next to gene or protein names indicate increased or decreased expression, respectively. ADAM, a disintegrin and metalloprotease; α-SMA, α-smooth muscle actin; CTGF, connective tissue growth factor; CSC, cancer stem cells; ECM, extracellular matrix; EMT, epithelial–mesenchymal transition; ERK, extracellular signal-regulated kinase; GABA_A_, gamma-aminobutyric acid subtype A; HSCs, hepatic stellate cells; JNK, c-Jun N-terminal kinases; MAPK, mitogen-activated protein kinase; mTOR, mammalian target of rapamycin; NF-κB, nuclear factor kappa B; PD-L1, programmed death-ligand 1; PI3K, phosphatidylinositol 3-kinase; TGF, transforming growth factor; YAP1, yes-associated protein 1. The figure was created in https://BioRender.com (accessed on 16 July 2025).

**Figure 2:**
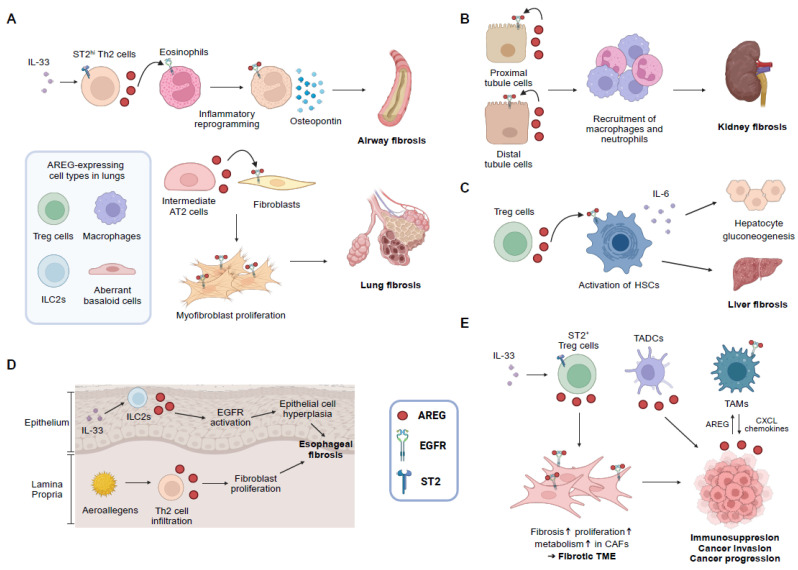
AREG-involved intercellular interactions that contribute to fibrosis formation and cancer progression. (**A**) AREG production by ST2^hi^ memory Th2 cells are induced by IL-33. AREG activates EGFR signaling in eosinophils. They are transcriptionally reprogrammed to secrete osteopontin, which is required for airway fibrosis. In fibrotic lungs, upregulated AREG in intermediate AT2 cells leads to lung fibrosis development. Treg cells, ILC2s, macrophages, and aberrant basaloid cells are known sources of AREG in fibrotic lungs. (**B**) Proximal and distal tubule-derived AREG activates EGFR signals. This recruits macrophages and neutrophils, which leads to renal inflammation and fibrosis. (**C**) Treg cells that are enriched in the diseased liver express AREG. It activates HSCs via EGFR signaling. Activated HSCs exacerbate liver fibrosis and promote gluconeogenesis in hepatocytes through IL-6. (**D**) Increased IL-33 in eosinophilic esophagitis recruited ILC2s at the epithelial border. AREG derived by ILC2s induced esophageal epithelial thickness and basal hyperplasia. Repeated house dust mite exposure accumulates AREG-producing Th2 cells in the lamina propria, in which AREG promotes esophageal fibroblast proliferation. (**E**) The alarmin IL-33 increases the accumulation of ST2^+^ Treg cells in tumors. Treg cells-derived AREG turns CAFs into profibrotic and proliferative states, which fosters a fibrotic TME and causes immunosuppression. ADAM17-mediated shedding of EGFR ligands, including AREG, by cancer cells promotes formation of TAMs. TAMs induce cancer cell invasion by secreting CXCL chemokines [243]. Moreover, TADCs-derived AREG increased cancer progression. The upward arrows (↑) next to the words indicate increased incidence of the phenomenon. AT2, alveolar type 2; CAFs, cancer-associated fibroblasts; CXCL, C-X-C motif chemokine ligand; EGFR, epidermal growth factor receptor; HSCs, hepatic stellate cells; IL, interleukin; ILC2s, type 2 innate lymphoid cells; ST2, IL-33 receptor; TADCs, tumor-associated dendritic cells; TAMs, tumor-associated macrophages; Th2, T helper 2; TME, tumor microenvironment; Treg, regulatory T. The figure was created in https://BioRender.com (accessed on 16 July 2025).

AREG has emerged as a central mediator in the pathophysiology of fibrosis and fibrosis-associated cancers across multiple organ systems, acting through both EGFR-dependent and integrin-mediated pathways and diverse intercellular interactions (Figure 1 and Figure 2). This review highlights how AREG’s unique signaling properties, such as its preferential activation of PLCγ via EGFR Tyr992 and its capacity to activate latent TGF-β through αvβ integrins, enable sustained fibrotic signaling in cell- and tissue-specific contexts [15,16]. In particular, the dual role of AREG, promoting repair or exacerbating fibrosis depending on its cellular origin and timing, underscores the complexity of its biology. These mechanistic nuances help explain the partial efficacy of conventional EGFR inhibitors and highlights the need for more precise, targeted antifibrotic and anticancer therapies.

Notably, the consistent upregulation of AREG in fibrotic and fibrosis-associated neoplastic conditions highlights a shared pathogenic axis between fibrosis and cancer. AREG-driven microenvironments facilitate both ECM remodeling and immune evasion, positioning AREG as a key node in the fibrotic–tumor continuum. This convergence offers a unique therapeutic window wherein AREG-targeted strategies could disrupt disease progression in both fibrosis and cancer simultaneously.

Moreover, variability in AREG expression based on genetic background and immune status reinforces the importance of patient stratification. The success of AREG inhibition will likely depend on the precise identification of AREG-producing and -responding cells, as well as the temporal dynamics of its expression. First, the precise temporal dynamics of AREG expression during disease progression remain incompletely characterized, limiting our ability to identify optimal therapeutic windows. Second, the relative contributions of EGFR-dependent versus integrin-mediated TGF-β activation pathways in different fibrotic contexts require further elucidation. Third, the molecular mechanisms that control the transition from reparative to pathological AREG signaling are still unknown. These findings of knowledge gaps argue for a personalized medicine approach to AREG-targeted therapy, integrating precise diagnostics with tailored intervention windows.

AREG is more than a downstream effector of EGFR signaling—it is a context-dependent regulator of fibrotic remodeling and a promising therapeutic target. The advent of novel strategies such as SAMiRNA-AREG offers new opportunities to intervene in both fibrotic and cancerous processes, with improved specificity and safety. SAMiRNA-AREG strategies demonstrate promising preclinical efficacy with favorable safety profiles, offering advantages over traditional siRNA therapeutics through reduced immunogenicity and enhanced targeting specificity. The drug targeting AREG could sensitize a fibrotic tumor to chemotherapy and immunotherapy by changing microenvironments, which will present the enhanced efficacy of combination therapies. However, successful clinical translation will require patient stratification based on AREG expression profiles and the careful consideration of cell-specific targeting approaches.

Future research should focus on several key areas: (1) mapping the spatiotemporal expression of AREG across disease stages; (2) elucidating cell-type specific effects in human fibrotic tissues using single-cell technologies; (3) defining mechanistic switches from reparative to fibrotic AREG signaling; (4) optimizing delivery systems for cell-specific AREG inhibition; (5) defining long-term effects and safety of AREG inhibition; and (6) advancing preclinical AREG inhibitors into clinical trials.

In conclusion, AREG represents both a promising therapeutic target and a valuable biomarker for fibrotic diseases and cancer. Its unique position at the intersection of tissue repair, fibrosis, and cancer progression offers unprecedented opportunities for developing integrated treatment strategies. As we understand more about the AREG function in fibrotic diseases and cancer, we can utilize its potential for therapeutics to transform these incurable fibrotic conditions into modifiable diseases.

## Abbreviations

The following abbreviations are used in this manuscript:

ADAM	A disintegrin and metalloprotease
AKI	Acute kidney injury
AREG	Amphiregulin
α-SMA	α-smooth muscle actin
CCl_4_	Carbon tetrachloride
CKD	Chronic kidney disease
DKD	Diabetic kidney disease
ECM	Extracellular matrix
EGF	Epidermal growth factor
EGFR	Epidermal growth factor receptor
EMT	Epithelial–mesenchymal transition
EoE	Eosinophilic esophagitis
ERK	Extracellular signal-regulated kinase
FPC	Fructose, palmitate, and cholesterol-rich
HB-EGF	Heparin-binding EGF-like growth factor
HSCs	Hepatic stellate cells
IBD	Inflammatory bowel disease
IIM	Inflammatory myopathy
IL	Interleukin
ILC2s	Type 2 innate lymphoid cells
IPF	Idiopathic pulmonary fibrosis
KO	Knockout
MAIT	Mucosal-associated invariant T
MAPK	Mitogen-activated protein kinase
MASH	Metabolic dysfunction-associated steatohepatitis
MNPs	Mononuclear phagocytes
PD-L1	Programmed death-ligand 1
PI3K	Phosphoinositide 3-kinase
PLCγ	Phospholipase C-γ
SAMiRNA	Self-Assembled-Micelle inhibitory RNA
SBRT	Stereotactic body radiotherapy
SSc	Systemic sclerosis
SSc-ILD	Systemic sclerosis-associated interstitial lung disease
TBI	Total-body irradiation
TGF	Transforming growth factor
Th	T helper
TKI	Tyrosine kinase inhibitor
TME	Tumor microenvironment

## References

[1] Wynn T.A. (2008). Cellular and molecular mechanisms of fibrosis. J. Pathol..

[2] Kisseleva T., Brenner D.A. (2008). Fibrogenesis of parenchymal organs. Proc. Am. Thorac. Soc..

[3] Miao H., Wu X.Q., Zhang D.D., Wang Y.N., Guo Y., Li P., Xiong Q., Zhao Y.Y. (2021). Deciphering the cellular mechanisms underlying fibrosis-associated diseases and therapeutic avenues. Pharmacol. Res..

[4] Lurje I., Gaisa N.T., Weiskirchen R., Tacke F. (2023). Mechanisms of organ fibrosis: Emerging concepts and implications for novel treatment strategies. Mol. Asp. Med..

[5] Kis K., Liu X., Hagood J.S. (2011). Myofibroblast differentiation and survival in fibrotic disease. Expert Rev. Mol. Med..

[6] Wynn T.A., Ramalingam T.R. (2012). Mechanisms of fibrosis: Therapeutic translation for fibrotic disease. Nat. Med..

[7] Dees C., Chakraborty D., Distler J.H.W. (2021). Cellular and molecular mechanisms in fibrosis. Exp. Dermatol..

[8] Umbarkar P., Ejantkar S., Tousif S., Lal H. (2021). Mechanisms of Fibroblast Activation and Myocardial Fibrosis: Lessons Learned from FB-Specific Conditional Mouse Models. Cells.

[9] Friedman S.L., Sheppard D., Duffield J.S., Violette S. (2013). Therapy for fibrotic diseases: Nearing the starting line. Sci. Transl. Med..

[10] Distler J.H., Feghali-Bostwick C., Soare A., Asano Y., Distler O., Abraham D.J. (2017). Review: Frontiers of Antifibrotic Therapy in Systemic Sclerosis. Arthritis Rheumatol..

[11] Zhao M., Wang L., Wang M., Zhou S., Lu Y., Cui H., Racanelli A.C., Zhang L., Ye T., Ding B. (2022). Targeting fibrosis, mechanisms and cilinical trials. Signal Transduct. Target. Ther..

[12] Zaiss D.M.W., Gause W.C., Osborne L.C., Artis D. (2015). Emerging functions of amphiregulin in orchestrating immunity, inflammation, and tissue repair. Immunity.

[13] Berasain C., Avila M.A. (2014). Amphiregulin. Semin. Cell Dev. Biol..

[14] Singh S.S., Chauhan S.B., Kumar A., Kumar S., Engwerda C.R., Sundar S., Kumar R. (2022). Amphiregulin in cellular physiology, health, and disease: Potential use as a biomarker and therapeutic target. J. Cell Physiol..

[15] Minutti C.M., Modak R.V., Macdonald F., Li F., Smyth D.J., Dorward D.A., Blair N., Husovsky C., Muir A., Giampazolias E. (2019). A Macrophage-Pericyte Axis Directs Tissue Restoration via Amphiregulin-Induced Transforming Growth Factor Beta Activation. Immunity.

[16] Zaiss D.M.W. (2020). Amphiregulin as a driver of tissue fibrosis. Am. J. Transplant..

[17] Shoyab M., McDonald V.L., Bradley J.G., Todaro G.J. (1988). Amphiregulin: A bifunctional growth-modulating glycoprotein produced by the phorbol 12-myristate 13-acetate-treated human breast adenocarcinoma cell line MCF-7. Proc. Natl. Acad. Sci. USA.

[18] Busser B., Sancey L., Brambilla E., Coll J.L., Hurbin A. (2011). The multiple roles of amphiregulin in human cancer. Biochim. Biophys. Acta.

[19] Gilmore J.L., Scott J.A., Bouizar Z., Robling A., Pitfield S.E., Riese D.J., Foley J. (2008). Amphiregulin-EGFR signaling regulates PTHrP gene expression in breast cancer cells. Breast Cancer Res. Treat..

[20] Stoll S.W., Johnson J.L., Bhasin A., Johnston A., Gudjonsson J.E., Rittie L., Elder J.T. (2010). Metalloproteinase-mediated, context-dependent function of amphiregulin and HB-EGF in human keratinocytes and skin. J. Investig. Dermatol..

[21] Kefaloyianni E., Muthu M.L., Kaeppler J., Sun X., Sabbisetti V., Chalaris A., Rose-John S., Wong E., Sagi I., Waikar S.S. (2016). ADAM17 substrate release in proximal tubule drives kidney fibrosis. JCI Insight.

[22] Stoll S.W., Stuart P.E., Lambert S., Gandarillas A., Rittie L., Johnston A., Elder J.T. (2016). Membrane-Tethered Intracellular Domain of Amphiregulin Promotes Keratinocyte Proliferation. J. Investig. Dermatol..

[23] Zaiss D.M., Minutti C.M., Knipper J.A. (2019). Immune- and non-immune-mediated roles of regulatory T-cells during wound healing. Immunology.

[24] Berasain C., Garcia-Trevijano E.R., Castillo J., Erroba E., Lee D.C., Prieto J., Avila M.A. (2005). Amphiregulin: An early trigger of liver regeneration in mice. Gastroenterology.

[25] Schelfhout V.R., Coene E.D., Delaey B., Waeytens A.A., De Rycke L., Deleu M., De Potter C.R. (2002). The role of heregulin-alpha as a motility factor and amphiregulin as a growth factor in wound healing. J. Pathol..

[26] Wang J., Li J., Wang S., Pan Y., Yang J., Yin L., Dou H., Hou Y. (2024). Amphiregulin secreted by umbilical cord multipotent stromal cells protects against ferroptosis of macrophages via the activating transcription factor 3-CD36 axis to alleviate endometrial fibrosis. Stem Cells.

[27] Liu T., Santos F., Ding L., Wu Z., Phan S. (2016). Amphiregulin Promotes Fibroblast Activation in Pulmonary Fibrosis. FASEB J..

[28] Cheng W.H., Kao S.Y., Chen C.L., Yuliani F.S., Lin L.Y., Lin C.H., Chen B.C. (2022). Amphiregulin induces CCN2 and fibronectin expression by TGF-beta through EGFR-dependent pathway in lung epithelial cells. Respir. Res..

[29] Zhou J., Xu Y., Li Y., Zhang Q., Zhong L., Pan W., Ji K., Zhang S., Chen Z., Liu Y. (2025). Cancer-associated fibroblasts derived amphiregulin promotes HNSCC progression and drug resistance of EGFR inhibitor. Cancer Lett..

[30] Zhao R., Wang Z., Wang G., Geng J., Wu H., Liu X., Bin E., Sui J., Dai H., Tang N. (2024). Sustained amphiregulin expression in intermediate alveolar stem cells drives progressive fibrosis. Cell Stem Cell.

[31] Son S.S., Hwang S., Park J.H., Ko Y., Yun S.I., Lee J.H., Son B., Kim T.R., Park H.O., Lee E.Y. (2021). In vivo silencing of amphiregulin by a novel effective Self-Assembled-Micelle inhibitory RNA ameliorates renal fibrosis via inhibition of EGFR signals. Sci. Rep..

[32] Son B., Kim T.R., Park J.H., Yun S.I., Choi H., Choi J.W., Jeon C., Park H.O. (2022). SAMiRNA Targeting Amphiregulin Alleviate Total-Body-Irradiation-Induced Renal Fibrosis. Radiat. Res..

[33] Yoon P.O., Park J.W., Lee C.M., Kim S.H., Kim H.N., Ko Y., Bae S.J., Yun S., Park J.H., Kwon T. (2016). Self-assembled Micelle Interfering RNA for Effective and Safe Targeting of Dysregulated Genes in Pulmonary Fibrosis. J. Biol. Chem..

[34] Xu Q., Chiao P., Sun Y. (2016). Amphiregulin in Cancer: New Insights for Translational Medicine. Trends Cancer.

[35] Xu Q., Long Q., Zhu D., Fu D., Zhang B., Han L., Qian M., Guo J., Xu J., Cao L. (2019). Targeting amphiregulin (AREG) derived from senescent stromal cells diminishes cancer resistance and averts programmed cell death 1 ligand (PD-L1)-mediated immunosuppression. Aging Cell.

[36] Baldys A., Gooz M., Morinelli T.A., Lee M.H., Raymond J.R., Luttrell L.M., Raymond J.R. (2009). Essential role of c-Cbl in amphiregulin-induced recycling and signaling of the endogenous epidermal growth factor receptor. Biochemistry.

[37] Krall J.A., Beyer E.M., MacBeath G. (2011). High- and low-affinity epidermal growth factor receptor-ligand interactions activate distinct signaling pathways. PLoS ONE.

[38] Tomasello C., Baldessari C., Napolitano M., Orsi G., Grizzi G., Bertolini F., Barbieri F., Cascinu S. (2018). Resistance to EGFR inhibitors in non-small cell lung cancer: Clinical management and future perspectives. Crit. Rev. Oncol. Hematol..

[39] Nele Van Der S., Elisa G., Daniela C., Alessandro L., Christian D.R., Godefridus J.P. (2018). Resistance to epidermal growth factor receptor inhibition in non-small cell lung cancer. Cancer Drug Resist..

[40] Wilson K.J., Gilmore J.L., Foley J., Lemmon M.A., Riese D.J. (2009). Functional selectivity of EGF family peptide growth factors: Implications for cancer. Pharmacol. Ther..

[41] Busser B., Sancey L., Josserand V., Niang C., Favrot M.C., Coll J.L., Hurbin A. (2010). Amphiregulin promotes BAX inhibition and resistance to gefitinib in non-small-cell lung cancers. Mol. Ther..

[42] Busser B., Sancey L., Josserand V., Niang C., Khochbin S., Favrot M.C., Coll J.L., Hurbin A. (2010). Amphiregulin promotes resistance to gefitinib in nonsmall cell lung cancer cells by regulating Ku70 acetylation. Mol. Ther..

[43] Macdonald-Obermann J.L., Pike L.J. (2014). Different epidermal growth factor (EGF) receptor ligands show distinct kinetics and biased or partial agonism for homodimer and heterodimer formation. J. Biol. Chem..

[44] Deguchi E., Lin S., Hirayama D., Matsuda K., Tanave A., Sumiyama K., Tsukiji S., Otani T., Furuse M., Sorkin A. (2024). Low-affinity ligands of the epidermal growth factor receptor are long-range signal transmitters in collective cell migration of epithelial cells. Cell Rep..

[45] Schramm F., Schaefer L., Wygrecka M. (2022). EGFR Signaling in Lung Fibrosis. Cells.

[46] Zhuang S., Liu N. (2014). EGFR signaling in renal fibrosis. Kidney Int. Suppl..

[47] Singh B., Carpenter G., Coffey R.J. (2016). EGF receptor ligands: Recent advances. F1000Research.

[48] Kefaloyianni E., Keerthi Raja M.R., Schumacher J., Muthu M.L., Krishnadoss V., Waikar S.S., Herrlich A. (2019). Proximal Tubule-Derived Amphiregulin Amplifies and Integrates Profibrotic EGF Receptor Signals in Kidney Fibrosis. J. Am. Soc. Nephrol..

[49] Khan Z., Marshall J.F. (2016). The role of integrins in TGFbeta activation in the tumour stroma. Cell Tissue Res..

[50] Hao Y., Baker D., Ten Dijke P. (2019). TGF-beta-Mediated Epithelial-Mesenchymal Transition and Cancer Metastasis. Int. J. Mol. Sci..

[51] Rexer B.N., Engelman J.A., Arteaga C.L. (2009). Overcoming resistance to tyrosine kinase inhibitors: Lessons learned from cancer cells treated with EGFR antagonists. Cell Cycle.

[52] Wheeler D.L., Dunn E.F., Harari P.M. (2010). Understanding resistance to EGFR inhibitors-impact on future treatment strategies. Nat. Rev. Clin. Oncol..

[53] Edgar K.A., Crocker L., Cheng E., Wagle M.C., Wongchenko M., Yan Y., Wilson T.R., Dompe N., Neve R.M., Belvin M. (2014). Amphiregulin and PTEN evoke a multimodal mechanism of acquired resistance to PI3K inhibition. Genes Cancer.

[54] Kindermann M., Knipfer L., Atreya I., Wirtz S. (2018). ILC2s in infectious diseases and organ-specific fibrosis. Semin. Immunopathol..

[55] Hirahara K., Aoki A., Morimoto Y., Kiuchi M., Okano M., Nakayama T. (2019). The immunopathology of lung fibrosis: Amphiregulin-producing pathogenic memory T helper-2 cells control the airway fibrotic responses by inducing eosinophils to secrete osteopontin. Semin. Immunopathol..

[56] Adams T.S., Schupp J.C., Poli S., Ayaub E.A., Neumark N., Ahangari F., Chu S.G., Raby B.A., DeIuliis G., Januszyk M. (2020). Single-cell RNA-seq reveals ectopic and aberrant lung-resident cell populations in idiopathic pulmonary fibrosis. Sci. Adv..

[57] Neumark N., Cosme C., Rose K.A., Kaminski N. (2020). The Idiopathic Pulmonary Fibrosis Cell Atlas. Am. J. Physiol. Lung Cell Mol. Physiol..

[58] Kathiriya J.J., Wang C., Zhou M., Brumwell A., Cassandras M., Le Saux C.J., Cohen M., Alysandratos K.D., Wang B., Wolters P. (2022). Human alveolar type 2 epithelium transdifferentiates into metaplastic KRT5^+^ basal cells. Nat. Cell Biol..

[59] Nakanishi T., Koma Y.I., Miyako S., Torigoe R., Yokoo H., Omori M., Yamanaka K., Ishihara N., Tsukamoto S., Kodama T. (2024). AREG Upregulation in Cancer Cells via Direct Interaction with Cancer-Associated Fibroblasts Promotes Esophageal Squamous Cell Carcinoma Progression Through EGFR-Erk/p38 MAPK Signaling. Cells.

[60] Buechler M.B., Fu W., Turley S.J. (2021). Fibroblast-macrophage reciprocal interactions in health, fibrosis, and cancer. Immunity.

[61] Ding L., Liu T., Wu Z., Hu B., Nakashima T., Ullenbruch M., Gonzalez De Los Santos F., Phan S.H. (2016). Bone Marrow CD11c^+^ Cell-Derived Amphiregulin Promotes Pulmonary Fibrosis. J. Immunol..

[62] Kurche J.S., Stancil I.T., Michalski J.E., Yang I.V., Schwartz D.A. (2022). Dysregulated Cell-Cell Communication Characterizes Pulmonary Fibrosis. Cells.

[63] Kaiser K.A., Loffredo L.F., Santos-Alexis K.L., Ringham O.R., Arpaia N. (2023). Regulation of the alveolar regenerative niche by amphiregulin-producing regulatory T cells. J. Exp. Med..

[64] Miyamoto S., Fukami T., Yagi H., Kuroki M., Yotsumoto F. (2009). Potential for molecularly targeted therapy against epidermal growth factor receptor ligands. Anticancer Res..

[65] Lofgren K.A., Sreekumar S., Jenkins E.C., Ernzen K.J., Kenny P.A. (2021). Anti-tumor efficacy of an MMAE-conjugated antibody targeting cell surface TACE/ADAM17-cleaved Amphiregulin in breast cancer. Antib. Ther..

[66] Hosur V., Farley M.L., Burzenski L.M., Shultz L.D., Wiles M.V. (2018). ADAM17 is essential for ectodomain shedding of the EGF-receptor ligand amphiregulin. FEBS Open Bio.

[67] Richards F.M., Tape C.J., Jodrell D.I., Murphy G. (2012). Anti-tumour effects of a specific anti-ADAM17 antibody in an ovarian cancer model in vivo. PLoS ONE.

[68] Dosch J., Ziemke E., Wan S., Luker K., Welling T., Hardiman K., Fearon E., Thomas S., Flynn M., Rios-Doria J. (2017). Targeting ADAM17 inhibits human colorectal adenocarcinoma progression and tumor-initiating cell frequency. Oncotarget.

[69] Lee C.M., Park J.W., Cho W.K., Zhou Y., Han B., Yoon P.O., Chae J., Elias J.A., Lee C.G. (2014). Modifiers of TGF-beta1 effector function as novel therapeutic targets of pulmonary fibrosis. Korean J. Intern. Med..

[70] Peng D., Fu M., Wang M., Wei Y., Wei X. (2022). Targeting TGF-beta signal transduction for fibrosis and cancer therapy. Mol. Cancer.

[71] Trachalaki A., Sultana N., Wells A.U. (2023). An update on current and emerging drug treatments for idiopathic pulmonary fibrosis. Expert. Opin. Pharmacother..

[72] Suri G.S., Kaur G., Jha C.K., Tiwari M. (2021). Understanding idiopathic pulmonary fibrosis—Clinical features, molecular mechanism and therapies. Exp. Gerontol..

[73] Zaman T., Lee J.S. (2018). Risk factors for the development of idiopathic pulmonary fibrosis: A review. Curr. Pulmonol. Rep..

[74] Zhang Y., Wang J. (2023). Cellular and Molecular Mechanisms in Idiopathic Pulmonary Fibrosis. Adv. Respir. Med..

[75] Bonella F., Spagnolo P., Ryerson C. (2023). Current and Future Treatment Landscape for Idiopathic Pulmonary Fibrosis. Drugs.

[76] Arshad M., Athar Z.M., Hiba T. (2024). Current and Novel Treatment Modalities of Idiopathic Pulmonary Fibrosis. Cureus.

[77] Cameli P., Refini R.M., Bergantini L., d’Alessandro M., Alonzi V., Magnoni C., Rottoli P., Sestini P., Bargagli E. (2020). Long-Term Follow-Up of Patients with Idiopathic Pulmonary Fibrosis Treated with Pirfenidone or Nintedanib: A Real-Life Comparison Study. Front. Mol. Biosci..

[78] Marijic P., Schwarzkopf L., Schwettmann L., Ruhnke T., Trudzinski F., Kreuter M. (2021). Pirfenidone vs. nintedanib in patients with idiopathic pulmonary fibrosis: A retrospective cohort study. Respir. Res..

[79] Kalluri M. (2024). Palliative care in advanced pulmonary fibrosis. Curr. Opin. Pulm. Med..

[80] Hanata N., Nagafuchi Y., Sugimori Y., Kobayashi S., Tsuchida Y., Iwasaki Y., Shoda H., Fujio K. (2021). Serum Amphiregulin and Heparin-Binding Epidermal Growth Factor as Biomarkers in Patients with Idiopathic Inflammatory Myopathy. J. Clin. Med..

[81] Shen C., Fan X., Mao Y., Jiang J. (2024). Amphiregulin in lung diseases: A review. Medicine.

[82] Shen M., Luo Z., Zhou Y. (2022). Regeneration-Associated Transitional State Cells in Pulmonary Fibrosis. Int. J. Mol. Sci..

[83] Yao C., Guan X., Carraro G., Parimon T., Liu X., Huang G., Mulay A., Soukiasian H.J., David G., Weigt S.S. (2021). Senescence of Alveolar Type 2 Cells Drives Progressive Pulmonary Fibrosis. Am. J. Respir. Crit. Care Med..

[84] Liu T., Ding L., Wu Z., Gonzalez De Los Santos F., Phan S. (2015). Role of dendritic cell-derived amphiregulin in pulmonary fibrosis (CCR5P.203). J. Immunol..

[85] Yao H.C., Zhu Y., Lu H.Y., Ju H.M., Xu S.Q., Qiao Y., Wei S.J. (2023). Type 2 innate lymphoid cell-derived amphiregulin regulates type II alveolar epithelial cell transdifferentiation in a mouse model of bronchopulmonary dysplasia. Int. Immunopharmacol..

[86] Morimoto Y., Hirahara K., Kiuchi M., Wada T., Ichikawa T., Kanno T., Okano M., Kokubo K., Onodera A., Sakurai D. (2018). Amphiregulin-Producing Pathogenic Memory T Helper 2 Cells Instruct Eosinophils to Secrete Osteopontin and Facilitate Airway Fibrosis. Immunity.

[87] Zhou Y., Lee J.Y., Lee C.M., Cho W.K., Kang M.J., Koff J.L., Yoon P.O., Chae J., Park H.O., Elias J.A. (2012). Amphiregulin, an epidermal growth factor receptor ligand, plays an essential role in the pathogenesis of transforming growth factor-beta-induced pulmonary fibrosis. J. Biol. Chem..

[88] Conroy K.P., Kitto L.J., Henderson N.C. (2016). alphav integrins: Key regulators of tissue fibrosis. Cell Tissue Res..

[89] Margadant C., Sonnenberg A. (2010). Integrin-TGF-beta crosstalk in fibrosis, cancer and wound healing. EMBO Rep..

[90] Sakao S., Tatsumi K. (2012). Molecular mechanisms of lung-specific toxicity induced by epidermal growth factor receptor tyrosine kinase inhibitors. Oncol. Lett..

[91] Fernandez I.E., Eickelberg O. (2012). The impact of TGF-beta on lung fibrosis: From targeting to biomarkers. Proc. Am. Thorac. Soc..

[92] Suzuki H., Aoshiba K., Yokohori N., Nagai A. (2003). Epidermal growth factor receptor tyrosine kinase inhibition augments a murine model of pulmonary fibrosis. Cancer Res..

[93] Ma H., Wu X., Li Y., Xia Y. (2022). Research Progress in the Molecular Mechanisms, Therapeutic Targets, and Drug Development of Idiopathic Pulmonary Fibrosis. Front. Pharmacol..

[94] Koya D. (2019). Diabetic kidney disease: Its current trends and future therapeutic perspectives. J. Diabetes Investig..

[95] Humphreys B.D. (2018). Mechanisms of Renal Fibrosis. Annu. Rev. Physiol..

[96] Liu B.C., Tang T.T., Lv L.L., Lan H.Y. (2018). Renal tubule injury: A driving force toward chronic kidney disease. Kidney Int..

[97] Klinkhammer B.M., Boor P. (2023). Kidney fibrosis: Emerging diagnostic and therapeutic strategies. Mol. Asp. Med..

[98] Huang R., Fu P., Ma L. (2023). Kidney fibrosis: From mechanisms to therapeutic medicines. Signal Transduct. Target. Ther..

[99] Melderis S., Hagenstein J., Warkotsch M.T., Dang J., Herrnstadt G.R., Niehus C.B., Neumann K., Panzer U., Berasain C., Avila M.A. (2020). Amphiregulin Aggravates Glomerulonephritis via Recruitment and Activation of Myeloid Cells. J. Am. Soc. Nephrol..

[100] Rayego-Mateos S., Rodrigues-Diez R., Morgado-Pascual J.L., Valentijn F., Valdivielso J.M., Goldschmeding R., Ruiz-Ortega M. (2018). Role of Epidermal Growth Factor Receptor (EGFR) and Its Ligands in Kidney Inflammation and Damage. Mediat. Inflamm..

[101] Osakabe Y., Taniguchi Y., Hamada Ode K., Shimamura Y., Inotani S., Nishikawa H., Matsumoto T., Horino T., Fujimoto S., Terada Y. (2024). Clinical significance of amphiregulin in patients with chronic kidney disease. Clin. Exp. Nephrol..

[102] Schmidt I.M., Kefalogianni E., Zhao R., Verma A., Sabbisetti V., Rahman M., Pradhan N., Srivastava A., He J., Chen J. (2025). Associations of Serum Amphiregulin Levels with Kidney Failure and Mortality: The Chronic Renal Insufficiency Cohort (CRIC). Kidney Med..

[103] Wang X., Chen J., Xu J., Xie J., Harris D.C.H., Zheng G. (2021). The Role of Macrophages in Kidney Fibrosis. Front. Physiol..

[104] Cao Q., Harris D.C., Wang Y. (2015). Macrophages in kidney injury, inflammation, and fibrosis. Physiology.

[105] Black L.M., Lever J.M., Agarwal A. (2019). Renal Inflammation and Fibrosis: A Double-edged Sword. J. Histochem. Cytochem..

[106] Wynn T.A., Vannella K.M. (2016). Macrophages in Tissue Repair, Regeneration, and Fibrosis. Immunity.

[107] Palau V., Pascual J., Soler M.J., Riera M. (2019). Role of ADAM17 in kidney disease. Am. J. Physiol. Ren. Physiol..

[108] Gu Y.Y., Liu X.S., Huang X.R., Yu X.Q., Lan H.Y. (2020). Diverse Role of TGF-beta in Kidney Disease. Front. Cell Dev. Biol..

[109] Higgins S.P., Tang Y., Higgins C.E., Mian B., Zhang W., Czekay R.P., Samarakoon R., Conti D.J., Higgins P.J. (2018). TGF-beta1/p53 signaling in renal fibrogenesis. Cell Signal.

[110] Zhang Y., Meng X.M., Huang X.R., Lan H.Y. (2018). The preventive and therapeutic implication for renal fibrosis by targetting TGF-beta/Smad3 signaling. Clin. Sci..

[111] Chen J., Harris R.C. (2016). Interaction of the EGF Receptor and the Hippo Pathway in the Diabetic Kidney. J. Am. Soc. Nephrol..

[112] Melderis S., Warkotsch M.T., Dang J., Hagenstein J., Ehnold L.I., Herrnstadt G.R., Niehus C.B., Feindt F.C., Kylies D., Puelles V.G. (2022). The Amphiregulin/EGFR axis protects from lupus nephritis via downregulation of pathogenic CD4^+^ T helper cell responses. J. Autoimmun..

[113] Buvall L., Menzies R.I., Williams J., Woollard K.J., Kumar C., Granqvist A.B., Fritsch M., Feliers D., Reznichenko A., Gianni D. (2022). Selecting the right therapeutic target for kidney disease. Front. Pharmacol..

[114] Tawengi M., Al-Dali Y., Tawengi A., Benter I.F., Akhtar S. (2024). Targeting the epidermal growth factor receptor (EGFR/ErbB) for the potential treatment of renal pathologies. Front. Pharmacol..

[115] Lee S.Y., Kim S.I., Choi M.E. (2015). Therapeutic targets for treating fibrotic kidney diseases. Transl. Res..

[116] Wang Q., Wang F., Li X., Ma Z., Jiang D. (2023). Quercetin inhibits the amphiregulin/EGFR signaling-mediated renal tubular epithelial-mesenchymal transition and renal fibrosis in obstructive nephropathy. Phytother. Res..

[117] Kisseleva T., Brenner D. (2021). Molecular and cellular mechanisms of liver fibrosis and its regression. Nat. Rev. Gastroenterol. Hepatol..

[118] Unalp-Arida A., Ruhl C.E. (2017). Liver fibrosis scores predict liver disease mortality in the United States population. Hepatology.

[119] Ng C.H., Lim W.H., Hui Lim G.E., Hao Tan D.J., Syn N., Muthiah M.D., Huang D.Q., Loomba R. (2023). Mortality Outcomes by Fibrosis Stage in Nonalcoholic Fatty Liver Disease: A Systematic Review and Meta-analysis. Clin. Gastroenterol. Hepatol..

[120] Savage T.M., Fortson K.T., de Los Santos-Alexis K., Oliveras-Alsina A., Rouanne M., Rae S.S., Gamarra J.R., Shayya H., Kornberg A., Cavero R. (2024). Amphiregulin from regulatory T cells promotes liver fibrosis and insulin resistance in non-alcoholic steatohepatitis. Immunity.

[121] McKee C., Sigala B., Soeda J., Mouralidarane A., Morgan M., Mazzoccoli G., Rappa F., Cappello F., Cabibi D., Pazienza V. (2015). Amphiregulin activates human hepatic stellate cells and is upregulated in non alcoholic steatohepatitis. Sci. Rep..

[122] Fujiwara A., Takemura K., Tanaka A., Matsumoto M., Katsuyama M., Okanoue T., Yamaguchi K., Itoh Y., Iwata K., Amagase K. (2024). Carfilzomib shows therapeutic potential for reduction of liver fibrosis by targeting hepatic stellate cell activation. Sci. Rep..

[123] Zhou X., Liu W.M., Sun H.Y., Peng Y., Huang R.J., Chen C.Y., Zhang H.D., Zhou S.A., Wu H.P., Tang D. (2025). Hepatocyte-derived liver progenitor-like cells attenuate liver cirrhosis via induction of apoptosis in hepatic stellate cells. Hepatol. Commun..

[124] Keam S.J. (2024). Resmetirom: First Approval. Drugs.

[125] Heo Y.J., Lee N., Choi S.E., Jeon J.Y., Han S.J., Kim D.J., Kang Y., Lee K.W., Kim H.J. (2023). Amphiregulin Induces iNOS and COX-2 Expression through NF-kappaB and MAPK Signaling in Hepatic Inflammation. Mediat. Inflamm..

[126] Hori M., Kita M., Torihashi S., Miyamoto S., Won K.J., Sato K., Ozaki H., Karaki H. (2001). Upregulation of iNOS by COX-2 in muscularis resident macrophage of rat intestine stimulated with LPS. Am. J. Physiol. Gastrointest. Liver Physiol..

[127] Choi Y., Lee M.K., Lim S.Y., Sung S.H., Kim Y.C. (2009). Inhibition of inducible NO synthase, cyclooxygenase-2 and interleukin-1beta by torilin is mediated by mitogen-activated protein kinases in microglial BV2 cells. Br. J. Pharmacol..

[128] Dashek R.J., Cunningham R.P., Taylor C.L., Alessi I., Diaz C., Meers G.M., Wheeler A.A., Ibdah J.A., Parks E.J., Yoshida T. (2024). Hepatocellular RECK as a Critical Regulator of Metabolic Dysfunction-associated Steatohepatitis Development. Cell Mol. Gastroenterol. Hepatol..

[129] Cuevas M.J., Tieppo J., Marroni N.P., Tunon M.J., Gonzalez-Gallego J. (2011). Suppression of amphiregulin/epidermal growth factor receptor signals contributes to the protective effects of quercetin in cirrhotic rats. J. Nutr..

[130] Berasain C., Garcia-Trevijano E.R., Castillo J., Erroba E., Santamaria M., Lee D.C., Prieto J., Avila M.A. (2005). Novel role for amphiregulin in protection from liver injury. J. Biol. Chem..

[131] Liu Q., Rehman H., Krishnasamy Y., Haque K., Schnellmann R.G., Lemasters J.J., Zhong Z. (2012). Amphiregulin stimulates liver regeneration after small-for-size mouse liver transplantation. Am. J. Transplant..

[132] Perugorria M.J., Latasa M.U., Nicou A., Cartagena-Lirola H., Castillo J., Goni S., Vespasiani-Gentilucci U., Zagami M.G., Lotersztajn S., Prieto J. (2008). The epidermal growth factor receptor ligand amphiregulin participates in the development of mouse liver fibrosis. Hepatology.

[133] Fujii T., Fuchs B.C., Yamada S., Lauwers G.Y., Kulu Y., Goodwin J.M., Lanuti M., Tanabe K.K. (2010). Mouse model of carbon tetrachloride induced liver fibrosis: Histopathological changes and expression of CD133 and epidermal growth factor. BMC Gastroenterol..

[134] Crespo I., San-Miguel B., Fernandez A., Ortiz de Urbina J., Gonzalez-Gallego J., Tunon M.J. (2015). Melatonin limits the expression of profibrogenic genes and ameliorates the progression of hepatic fibrosis in mice. Transl. Res..

[135] Ikeno Y., Ohara D., Takeuchi Y., Watanabe H., Kondoh G., Taura K., Uemoto S., Hirota K. (2020). Foxp3+ Regulatory T Cells Inhibit CCl_4_-Induced Liver Inflammation and Fibrosis by Regulating Tissue Cellular Immunity. Front. Immunol..

[136] Zeng J., Fan J., Zhou H. (2023). Bile acid-mediated signaling in cholestatic liver diseases. Cell Biosci..

[137] Santamaria E., Rodriguez-Ortigosa C.M., Uriarte I., Latasa M.U., Urtasun R., Alvarez-Sola G., Barcena-Varela M., Colyn L., Arcelus S., Jimenez M. (2019). The Epidermal Growth Factor Receptor Ligand Amphiregulin Protects from Cholestatic Liver Injury and Regulates Bile Acids Synthesis. Hepatology.

[138] Xiao M.H., Wu S., Liang P., Ma D., Zhang J., Chen H., Zhong Z., Liu J., Jiang H., Feng X. (2024). Mucosal-associated invariant T cells promote ductular reaction through amphiregulin in biliary atresia. eBioMedicine.

[139] Mohagheghi S., Geramizadeh B., Nikeghbalian S., Khodadadi I., Karimi J., Khajehahmadi Z., Gharekhanloo F., Tavilani H. (2019). Intricate role of yes-associated protein1 in human liver cirrhosis: TGF-β1 still is a giant player. IUBMB Life.

[140] Frangogiannis N.G. (2021). Cardiac fibrosis. Cardiovasc. Res..

[141] Hinderer S., Schenke-Layland K. (2019). Cardiac fibrosis—A short review of causes and therapeutic strategies. Adv. Drug Deliv. Rev..

[142] Yin X., Yin X., Pan X., Zhang J., Fan X., Li J., Zhai X., Jiang L., Hao P., Wang J. (2023). Post-myocardial infarction fibrosis: Pathophysiology, examination, and intervention. Front. Pharmacol..

[143] Talman V., Ruskoaho H. (2016). Cardiac fibrosis in myocardial infarction-from repair and remodeling to regeneration. Cell Tissue Res..

[144] Schlittler M., Pramstaller P.P., Rossini A., De Bortoli M. (2023). Myocardial Fibrosis in Hypertrophic Cardiomyopathy: A Perspective from Fibroblasts. Int. J. Mol. Sci..

[145] Ho C.Y., Lopez B., Coelho-Filho O.R., Lakdawala N.K., Cirino A.L., Jarolim P., Kwong R., Gonzalez A., Colan S.D., Seidman J.G. (2010). Myocardial fibrosis as an early manifestation of hypertrophic cardiomyopathy. N. Engl. J. Med..

[146] Nguyen T.P., Qu Z., Weiss J.N. (2014). Cardiac fibrosis and arrhythmogenesis: The road to repair is paved with perils. J. Mol. Cell Cardiol..

[147] Fujiu K., Shibata M., Nakayama Y., Ogata F., Matsumoto S., Noshita K., Iwami S., Nakae S., Komuro I., Nagai R. (2017). A heart-brain-kidney network controls adaptation to cardiac stress through tissue macrophage activation. Nat. Med..

[148] Sugita J., Fujiu K., Nakayama Y., Matsubara T., Matsuda J., Oshima T., Liu Y., Maru Y., Hasumi E., Kojima T. (2021). Cardiac macrophages prevent sudden death during heart stress. Nat. Commun..

[149] Koeppen M., Lee J.W., Seo S.W., Brodsky K.S., Kreth S., Yang I.V., Buttrick P.M., Eckle T., Eltzschig H.K. (2018). Hypoxia-inducible factor 2-alpha-dependent induction of amphiregulin dampens myocardial ischemia-reperfusion injury. Nat. Commun..

[150] Bu J., Huang S., Wang J., Xia T., Liu H., You Y., Wang Z., Liu K. (2021). The GABA(A) Receptor Influences Pressure Overload-Induced Heart Failure by Modulating Macrophages in Mice. Front. Immunol..

[151] Zuo C., Li X., Huang J., Chen D., Ji K., Yang Y., Xu T., Zhu D., Yan C., Gao P. (2018). Osteoglycin attenuates cardiac fibrosis by suppressing cardiac myofibroblast proliferation and migration through antagonizing lysophosphatidic acid 3/matrix metalloproteinase 2/epidermal growth factor receptor signalling. Cardiovasc. Res..

[152] Humeres C., Shinde A.V., Hanna A., Alex L., Hernandez S.C., Li R., Chen B., Conway S.J., Frangogiannis N.G. (2022). Smad7 effects on TGF-beta and ErbB2 restrain myofibroblast activation and protect from postinfarction heart failure. J. Clin. Investig..

[153] Grzebyk E., Pazgan-Simon M., Jagas J., Zuwala-Jagiello J., Gorka-Dynysiewicz J. (2022). Left ventricular function is related with amphiregulin and fibrosis markers in cirrhotic cardiomyopathy. J. Physiol. Pharmacol..

[154] Ji M., Liu Y., Zuo Z., Xu C., Lin L., Li Y. (2022). Downregulation of amphiregulin improves cardiac hypertrophy via attenuating oxidative stress and apoptosis. Biol. Direct.

[155] Warunek J.J., Fan L., Zhang X., Wang S., Sanders S.M., Li T., Mathews L.R., Dwyer G.K., Wood-Trageser M.A., Traczek S. (2024). Dysregulated Treg repair responses lead to chronic rejection after heart transplantation. J. Clin. Investig..

[156] Suzuki Y., Emoto T., Sato S., Yoshida T., Shoda M., Endoh H., Nagao M., Hamana T., Inoue T., Hayashi T. (2024). Left atrial single-cell transcriptomics reveals amphiregulin as a surrogate marker for atrial fibrillation. Commun. Biol..

[157] Porvari K., Horioka K., Kaija H., Pakanen L. (2024). Amphiregulin is overexpressed in human cardiac tissue in hypothermia deaths; associations between the transcript and stress hormone levels in cardiac deaths. Ann. Med..

[158] Monticelli L.A., Osborne L.C., Noti M., Tran S.V., Zaiss D.M., Artis D. (2015). IL-33 promotes an innate immune pathway of intestinal tissue protection dependent on amphiregulin-EGFR interactions. Proc. Natl. Acad. Sci. USA.

[159] Irie E., Ishihara R., Mizushima I., Hatai S., Hagihara Y., Takada Y., Tsunoda J., Iwata K., Matsubara Y., Yoshimatsu Y. (2022). Enrichment of type I interferon signaling in colonic group 2 innate lymphoid cells in experimental colitis. Front. Immunol..

[160] Shao J., Sheng H. (2010). Amphiregulin promotes intestinal epithelial regeneration: Roles of intestinal subepithelial myofibroblasts. Endocrinology.

[161] Wu J., Zhou B., Pang X., Song X., Gu Y., Xie R., Liu T., Xu X., Wang B., Cao H. (2022). Clostridium butyricum, a butyrate-producing potential probiotic, alleviates experimental colitis through epidermal growth factor receptor activation. Food Funct..

[162] Xiu W., Chen Q., Wang Z., Wang J., Zhou Z. (2020). Microbiota-derived short chain fatty acid promotion of Amphiregulin expression by dendritic cells is regulated by GPR43 and Blimp-1. Biochem. Biophys. Res. Commun..

[163] Zeka F., Angori S., Rutishauser D., Moch H., Posovszky C., Amin K., Holtan S., Gungor T., Drozdov D. (2025). High Amphiregulin Expression in Intestinal Biopsies of Pediatric Patients with Severe Acute Graft-Versus-Host Disease. Transplant. Cell Ther..

[164] D’Alessio S., Ungaro F., Noviello D., Lovisa S., Peyrin-Biroulet L., Danese S. (2022). Revisiting fibrosis in inflammatory bowel disease: The gut thickens. Nat. Rev. Gastroenterol. Hepatol..

[165] Alfredsson J., Wick M.J. (2020). Mechanism of fibrosis and stricture formation in Crohn’s disease. Scand. J. Immunol..

[166] Santacroce G., Lenti M.V., Di Sabatino A. (2022). Therapeutic Targeting of Intestinal Fibrosis in Crohn’s Disease. Cells.

[167] Wang Y., Huang B., Jin T., Ocansey D.K.W., Jiang J., Mao F. (2022). Intestinal Fibrosis in Inflammatory Bowel Disease and the Prospects of Mesenchymal Stem Cell Therapy. Front. Immunol..

[168] Zhao X., Yang W., Yu T., Yu Y., Cui X., Zhou Z., Yang H., Yu Y., Bilotta A.J., Yao S. (2023). Th17 Cell-Derived Amphiregulin Promotes Colitis-Associated Intestinal Fibrosis Through Activation of mTOR and MEK in Intestinal Myofibroblasts. Gastroenterology.

[169] Abdel-Wahab M., Giammarile F., Carrara M., Paez D., Hricak H., Ayati N., Li J.J., Mueller M., Aggarwal A., Al-Ibraheem A. (2024). Radiotherapy and theranostics: A Lancet Oncology Commission. Lancet Oncol..

[170] Fijardo M., Kwan J.Y.Y., Bissey P.A., Citrin D.E., Yip K.W., Liu F.F. (2024). The clinical manifestations and molecular pathogenesis of radiation fibrosis. EBioMedicine.

[171] Meulenbroeks C., van Weelden H., Schwartz C., Voehringer D., Redegeld F.A.M., Rutten V., Willemse T., Sijts A., Zaiss D.M.W. (2015). Basophil-derived amphiregulin is essential for UVB irradiation-induced immune suppression. J. Investig. Dermatol..

[172] Allanore Y., Simms R., Distler O., Trojanowska M., Pope J., Denton C.P., Varga J. (2015). Systemic sclerosis. Nat. Rev. Dis. Primers.

[173] Zhang M.Y., Fang S., Gao H., Zhang X., Gu D., Liu Y., Wan J., Xie J. (2021). A critical role of AREG for bleomycin-induced skin fibrosis. Cell Biosci..

[174] Padilla C.M., Valenzi E., Tabib T., Nazari B., Sembrat J., Rojas M., Fuschiotti P., Lafyatis R. (2024). Increased CD8^+^ tissue resident memory T cells, regulatory T cells and activated natural killer cells in systemic sclerosis lungs. Rheumatology.

[175] Morina G., Sambataro D., Libra A., Palmucci S., Colaci M., La Rocca G., Ferro F., Carli L., Baldini C., Liuzzo S.V. (2025). Recognition of Idiopathic Inflammatory Myopathies Underlying Interstitial Lung Diseases. Diagnostics.

[176] Ceribelli A., Tonutti A., Isailovic N., De Santis M., Selmi C. (2023). Interstitial lung disease associated with inflammatory myositis: Autoantibodies, clinical phenotypes, and progressive fibrosis. Front. Med..

[177] Sehgal S., Patel A., Chatterjee S., Fernandez A.P., Farver C., Yadav R., Li Y., Danoff S.K., Saygin D., Huapaya J.A. (2025). Idiopathic inflammatory myopathies related lung disease in adults. Lancet Respir. Med..

[178] Layoun H., Hajal J., Saliba Y., Smayra V., Habr B., Fares N. (2022). Pirfenidone mitigates TGF-β1-mediated fibrosis in an idiopathic inflammatory myositis-associated interstitial lung disease model. Cytokine.

[179] Lim M., Kim T., Kim H., Jang B.G., Myung J.K., Kim H.Y. (2025). Esophageal ILC2s mediate abnormal epithelial remodeling in eosinophilic esophagitis via Areg-EGFR signaling. Cell Mol. Immunol..

[180] Kaneko T., Iwamura C., Kiuchi M., Kurosugi A., Onoue M., Matsumura T., Chiba T., Nakayama T., Kato N., Hirahara K. (2024). Amphiregulin-producing T(H)2 cells facilitate esophageal fibrosis of eosinophilic esophagitis. J. Allergy Clin. Immunol. Glob..

[181] Ebott J., McAdams J., Kim C., Jansen C., Woodman M., De La Cruz P., Schrol C., Ribeiro J., James N. (2024). Enhanced amphiregulin exposure promotes modulation of the high grade serous ovarian cancer tumor immune microenvironment. Front. Pharmacol..

[182] Ishikawa N., Daigo Y., Takano A., Taniwaki M., Kato T., Hayama S., Murakami H., Takeshima Y., Inai K., Nishimura H. (2005). Increases of amphiregulin and transforming growth factor-alpha in serum as predictors of poor response to gefitinib among patients with advanced non-small cell lung cancers. Cancer Res..

[183] Masago K., Fujita S., Hatachi Y., Fukuhara A., Sakuma K., Ichikawa M., Kim Y.H., Mio T., Mishima M. (2008). Clinical significance of pretreatment serum amphiregulin and transforming growth factor-alpha, and an epidermal growth factor receptor somatic mutation in patients with advanced non-squamous, non-small cell lung cancer. Cancer Sci..

[184] Hobor S., Van Emburgh B.O., Crowley E., Misale S., Di Nicolantonio F., Bardelli A. (2014). TGFalpha and amphiregulin paracrine network promotes resistance to EGFR blockade in colorectal cancer cells. Clin. Cancer Res..

[185] Kappler C.S., Guest S.T., Irish J.C., Garrett-Mayer E., Kratche Z., Wilson R.C., Ethier S.P. (2015). Oncogenic signaling in amphiregulin and EGFR-expressing PTEN-null human breast cancer. Mol. Oncol..

[186] Higginbotham J.N., Demory Beckler M., Gephart J.D., Franklin J.L., Bogatcheva G., Kremers G.J., Piston D.W., Ayers G.D., McConnell R.E., Tyska M.J. (2011). Amphiregulin exosomes increase cancer cell invasion. Curr. Biol..

[187] Carvalho S., Lindzen M., Lauriola M., Shirazi N., Sinha S., Abdul-Hai A., Levanon K., Korach J., Barshack I., Cohen Y. (2016). An antibody to amphiregulin, an abundant growth factor in patients’ fluids, inhibits ovarian tumors. Oncogene.

[188] Khambata-Ford S., Garrett C.R., Meropol N.J., Basik M., Harbison C.T., Wu S., Wong T.W., Huang X., Takimoto C.H., Godwin A.K. (2007). Expression of epiregulin and amphiregulin and K-ras mutation status predict disease control in metastatic colorectal cancer patients treated with cetuximab. J. Clin. Oncol..

[189] Kim S.A., Park H., Kim K.J., Kim J.W., Sung J.H., Nam M., Lee J.H., Jung E.H., Suh K.J., Lee J.Y. (2021). Amphiregulin can predict treatment resistance to palliative first-line cetuximab plus FOLFIRI chemotherapy in patients with RAS wild-type metastatic colorectal cancer. Sci. Rep..

[190] Wang L., Wang L., Zhang H., Lu J., Zhang Z., Wu H., Liang Z. (2020). AREG mediates the epithelial-mesenchymal transition in pancreatic cancer cells via the EGFR/ERK/NF-kappaB signalling pathway. Oncol. Rep..

[191] Xiao B., Wang S., Pan Y., Zhi W., Gu C., Guo T., Zhai J., Li C., Chen Y.Q., Wang R. (2025). Development, opportunities, and challenges of siRNA nucleic acid drugs. Mol. Ther. Nucleic Acids.

[192] Piersma B., Hayward M.K., Weaver V.M. (2020). Fibrosis and cancer: A strained relationship. Biochim. Biophys. Acta Rev. Cancer.

[193] Landolt L., Spagnoli G.C., Hertig A., Brocheriou I., Marti H.P. (2022). Fibrosis and cancer: Shared features and mechanisms suggest common targeted therapeutic approaches. Nephrol. Dial. Transplant..

[194] Henke E., Nandigama R., Ergun S. (2019). Extracellular Matrix in the Tumor Microenvironment and Its Impact on Cancer Therapy. Front. Mol. Biosci..

[195] Benelli R., Vene R., Minghelli S., Carlone S., Gatteschi B., Ferrari N. (2013). Celecoxib induces proliferation and Amphiregulin production in colon subepithelial myofibroblasts, activating erk1-2 signaling in synergy with EGFR. Cancer Lett..

[196] Mucciolo G., Araos Henriquez J., Jihad M., Pinto Teles S., Manansala J.S., Li W., Ashworth S., Lloyd E.G., Cheng P.S.W., Luo W. (2024). EGFR-activated myofibroblasts promote metastasis of pancreatic cancer. Cancer Cell.

[197] Wu F., Yang J., Liu J., Wang Y., Mu J., Zeng Q., Deng S., Zhou H. (2021). Signaling pathways in cancer-associated fibroblasts and targeted therapy for cancer. Signal Transduct. Target. Ther..

[198] Chen Y., Huo R., Kang W., Liu Y., Zhao Z., Fu W., Ma R., Zhang X., Tang J., Zhu Z. (2023). Tumor-associated monocytes promote mesenchymal transformation through EGFR signaling in glioma. Cell Rep. Med..

[199] Jeong B.Y., Cho K.H., Jeong K.J., Cho S.J., Won M., Kim S.H., Cho N.H., Hur G.M., Yoon S.H., Park H.W. (2022). Lysophosphatidic acid-induced amphiregulin secretion by cancer-associated fibroblasts augments cancer cell invasion. Cancer Lett..

[200] Sun R., Zhao H., Gao D.S., Ni A., Li H., Chen L., Lu X., Chen K., Lu B. (2023). Amphiregulin couples IL1RL1^+^ regulatory T cells and cancer-associated fibroblasts to impede antitumor immunity. Sci. Adv..

[201] Wang S., Zhang Y., Wang Y., Ye P., Li J., Li H., Ding Q., Xia J. (2016). Amphiregulin Confers Regulatory T Cell Suppressive Function and Tumor Invasion via the EGFR/GSK-3beta/Foxp3 Axis. J. Biol. Chem..

[202] Hsu Y.L., Huang M.S., Cheng D.E., Hung J.Y., Yang C.J., Chou S.H., Kuo P.L. (2011). Lung tumor-associated dendritic cell-derived amphiregulin increased cancer progression. J. Immunol..

[203] Ballester B., Milara J., Cortijo J. (2019). Idiopathic Pulmonary Fibrosis and Lung Cancer: Mechanisms and Molecular Targets. Int. J. Mol. Sci..

[204] Yamaoka T., Ohba M., Ohmori T. (2017). Molecular-Targeted Therapies for Epidermal Growth Factor Receptor and Its Resistance Mechanisms. Int. J. Mol. Sci..

[205] Chandler C., Liu T., Buckanovich R., Coffman L.G. (2019). The double edge sword of fibrosis in cancer. Transl. Res..

[206] Jiang H., Hegde S., DeNardo D.G. (2017). Tumor-associated fibrosis as a regulator of tumor immunity and response to immunotherapy. Cancer Immunol. Immunother..

[207] Akhurst R.J., Hata A. (2012). Targeting the TGFbeta signalling pathway in disease. Nat. Rev. Drug Discov..

[208] Colak S., Ten Dijke P. (2017). Targeting TGF-beta Signaling in Cancer. Trends Cancer.

[209] Hawinkels L.J., Ten Dijke P. (2011). Exploring anti-TGF-beta therapies in cancer and fibrosis. Growth Factors.

[210] Huynh L.K., Hipolito C.J., Ten Dijke P. (2019). A Perspective on the Development of TGF-beta Inhibitors for Cancer Treatment. Biomolecules.

[211] Neuzillet C., Tijeras-Raballand A., Cohen R., Cros J., Faivre S., Raymond E., de Gramont A. (2015). Targeting the TGFbeta pathway for cancer therapy. Pharmacol. Ther..

[212] Ong C.H., Tham C.L., Harith H.H., Firdaus N., Israf D.A. (2021). TGF-beta-induced fibrosis: A review on the underlying mechanism and potential therapeutic strategies. Eur. J. Pharmacol..

[213] Guernsey-Biddle C., High P., Carmon K.S. (2024). Exploring the Potential of Epiregulin and Amphiregulin as Prognostic, Predictive, and Therapeutic Targets in Colorectal Cancer. Onco.

[214] Huang W.S., Wu K.L., Chen C.N., Chang S.F., Lee D.Y., Lee K.C. (2024). Amphiregulin Upregulation in Visfatin-Stimulated Colorectal Cancer Cells Reduces Sensitivity to 5-Fluororacil Cytotoxicity. Biology.

[215] Nagathihalli N.S., Beesetty Y., Lee W., Washington M.K., Chen X., Lockhart A.C., Merchant N.B. (2014). Novel mechanistic insights into ectodomain shedding of EGFR Ligands Amphiregulin and TGF-alpha: Impact on gastrointestinal cancers driven by secondary bile acids. Cancer Res..

[216] Li H., Fang R., Ma R., Long Y., He R., Lyu H., Chen L., Wen Y. (2024). Amphiregulin promotes activated regulatory T cell-suppressive function via the AREG/EGFR pathway in laryngeal squamous cell carcinoma. Head. Face Med..

[217] Jiang Y.J., Ho T.L., Chao C.C., He X.Y., Chen P.C., Cheng F.J., Huang W.C., Huang C.L., Liu P.I., Tang C.H. (2024). Particulate matter facilitates amphiregulin-dependent lung cancer proliferation through glutamine metabolism. Int. J. Biol. Sci..

[218] Tu C.Y., Wang B.W., Cheng F.J., Chen C.H., Hsia T.C., Wei Y.L., Chen C.Y., Hsieh I.S., Yeh Y.L., Wang L.Y. (2018). Incense burning smoke sensitizes lung cancer cells to EGFR TKI by inducing AREG expression. Am. J. Cancer Res..

[219] Piffko A., Yang K., Panda A., Heide J., Tesak K., Wen C., Zawieracz K., Wang L., Naccasha E.Z., Bugno J. (2025). Radiation-induced amphiregulin drives tumour metastasis. Nature.

[220] Thul P.J., Lindskog C. (2018). The human protein atlas: A spatial map of the human proteome. Protein Sci..

[221] Luetteke N.C., Qiu T.H., Fenton S.E., Troyer K.L., Riedel R.F., Chang A., Lee D.C. (1999). Targeted inactivation of the EGF and amphiregulin genes reveals distinct roles for EGF receptor ligands in mouse mammary gland development. Development.

[222] Jay F.F., Vaidya M., Porada S.M., Andrukhova O., Schneider M.R., Erben R.G. (2015). Amphiregulin lacks an essential role for the bone anabolic action of parathyroid hormone. Mol. Cell Endocrinol..

[223] Ciarloni L., Mallepell S., Brisken C. (2007). Amphiregulin is an essential mediator of estrogen receptor alpha function in mammary gland development. Proc. Natl. Acad. Sci. USA.

[224] Veit M., Ahrens B., Seidel J., Sommer A., Bhakdi S., Reiss K. (2019). Mutagenesis of the ADAM17-phosphatidylserine-binding motif leads to embryonic lethality in mice. Life Sci. Alliance.

[225] Kim T.R., Kim H.Y., Kim I.H., Kim K.C., Ko Y., Park J.H., Yun S., Lee I.C., Kim S.H., Park H.O. (2021). Safety pharmacology of self-assembled-micelle inhibitory RNA-targeting amphiregulin (SAMiRNA-AREG), a novel siRNA nanoparticle platform. Toxicol. Rep..

[226] Kim H.Y., Kim T.R., Kim S.H., Kim I.H., Lim J.O., Park J.H., Yun S., Lee I.C., Park H.O., Kim J.C. (2021). Four-Week Repeated Intravenous Dose Toxicity of Self-Assembled-Micelle Inhibitory RNA-Targeting Amphiregulin in Mice. Int. J. Toxicol..

[227] Kim H.Y., Kim T.R., Kim S.H., Kim I.H., Ko Y., Yun S., Lee I.C., Park H.O., Kim J.C. (2022). Genotoxicity evaluation of self-assembled-micelle inhibitory RNA-targeting amphiregulin (SAMiRNA-AREG), a novel siRNA nanoparticle for the treatment of fibrotic disease. Drug Chem. Toxicol..

[228] Kim H.-Y., Kim T.-R., Kim S.-H., Kim I.-H., Kim W.-I., Park J.-H., Ko Y., Yun S., Park H.-O., Kim J.-C. (2025). Systemic toxicity and toxicokinetics study of self-assembled-micelle inhibitory RNA-targeting amphiregulin in cynomolgus monkeys following intravenous injection. Toxicol. Res..

[229] Ranasinghe P., Addison M.L., Dear J.W., Webb D.J. (2023). Small interfering RNA: Discovery, pharmacology and clinical development-An introductory review. Br. J. Pharmacol..

[230] Gavrilov K., Saltzman W.M. (2012). Therapeutic siRNA: Principles, challenges, and strategies. Yale J. Biol. Med..

[231] Judge A., MacLachlan I. (2008). Overcoming the innate immune response to small interfering RNA. Hum. Gene Ther..

[232] Robbins M., Judge A., MacLachlan I. (2009). siRNA and innate immunity. Oligonucleotides.

[233] Sioud M. (2008). Does the understanding of immune activation by RNA predict the design of safe siRNAs?. Front. Biosci..

[234] Bora R.S., Gupta D., Mukkur T.K., Saini K.S. (2012). RNA interference therapeutics for cancer: Challenges and opportunities (review). Mol. Med. Rep..

[235] Deng Y., Wang C.C., Choy K.W., Du Q., Chen J., Wang Q., Li L., Chung T.K., Tang T. (2014). Therapeutic potentials of gene silencing by RNA interference: Principles, challenges, and new strategies. Gene.

[236] Sajid M.I., Moazzam M., Kato S., Yeseom Cho K., Tiwari R.K. (2020). Overcoming Barriers for siRNA Therapeutics: From Bench to Bedside. Pharmaceuticals.

[237] Yun S.I., Lee S.K., Goh E.A., Kwon O.S., Choi W., Kim J., Lee M.S., Choi S.J., Lim S.S., Moon T.K. (2022). Weekly treatment with SAMiRNA targeting the androgen receptor ameliorates androgenetic alopecia. Sci. Rep..

[238] Ali Zaidi S.S., Fatima F., Ali Zaidi S.A., Zhou D., Deng W., Liu S. (2023). Engineering siRNA therapeutics: Challenges and strategies. J. Nanobiotechnol..

[239] Kaushal A. (2023). Innate immune regulations and various siRNA modalities. Drug Deliv. Transl. Res..

[240] Carpenter B.L., Chen M., Knifley T., Davis K.A., Harrison S.M.W., Stewart R.L., O’Connor K.L. (2015). Integrin α6β4 Promotes Autocrine Epidermal Growth Factor Receptor (EGFR) Signaling to Stimulate Migration and Invasion toward Hepatocyte Growth Factor (HGF). J. Biol. Chem..

[241] Chen J.C., Chen Y.J., Lin C.Y., Fong Y.C., Hsu C.J., Tsai C.H., Su J.L., Tang C.H. (2015). Amphiregulin enhances α6β1 integrin expression and cell motility in human chondrosarcoma cells through Ras/Raf/MEK/ERK/AP-1 pathway. Oncotarget.

[242] Carpenter B.L., Liu J., Qi L., Wang C., O’Connor K.L. (2017). Integrin α6β4 Upregulates Amphiregulin and Epiregulin through Base Excision Repair-Mediated DNA Demethylation and Promotes Genome-wide DNA Hypomethylation. Sci. Rep..

[243] Gnosa S.P., Puig Blasco L., Piotrowski K.B., Freiberg M.L., Savickas S., Madsen D.H., Auf dem Keller U., Kronqvist P., Kveiborg M. (2022). ADAM17-mediated EGFR ligand shedding directs macrophage-promoted cancer cell invasion. JCI Insight.

